# Effects of Surrogate Hybridization and Adaptive Sampling for Simulation-Based Optimization

**DOI:** 10.1021/acs.iecr.4c03303

**Published:** 2025-04-15

**Authors:** Suryateja Ravutla, Andrew Bai, Matthew J. Realff, Fani Boukouvala

**Affiliations:** Department of Chemical and Biomolecular Engineering, Georgia Institute of Technology, Atlanta, Georgia 30332, United States

## Abstract

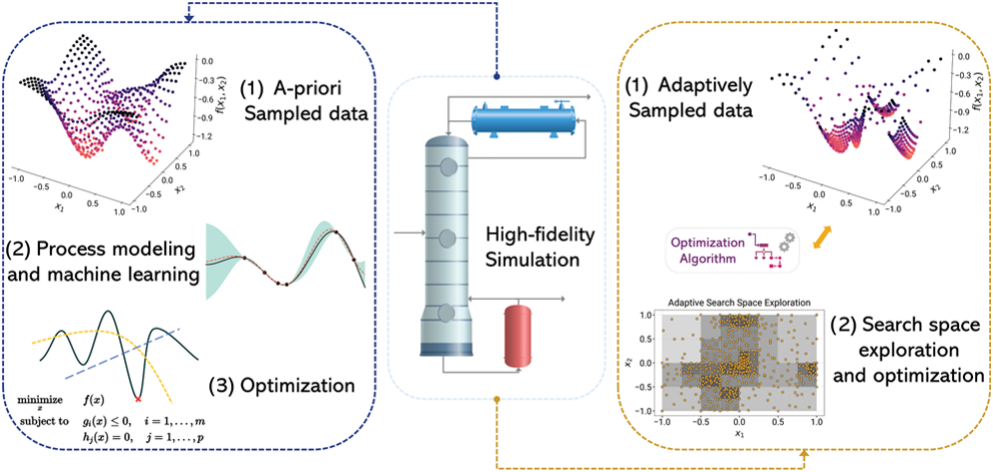

Process simulators are essential for modeling of complex processes; however, optimization of expensive models remains challenging due to lack of equations, simulation cost, and lack of convergence guarantees. To tackle these challenges, surrogate modeling and surrogate-based optimization methods have been proposed. Most commonly, surrogates are treated as black-box models, while recently hybrid surrogates have gained popularity. In this work, we assess two main methodologies: (a) optimization of surrogates trained using a set of fixed *a priori* samples using deterministic solvers, and (b) adaptive sampling-based optimization, which leverages surrogate predictions to guide the search process. Across both methods, we systematically compare the effect of black-box versus hybrid surrogates, that utilize a “model-correction” architecture combining different fidelity data. Through mathematical benchmarks with up to ten dimensions, and two engineering case studies for process design of an extractive distillation simulation model and an adsorption simulation model, we present the effects of sampling quantity, dimensionality, formulation, and hybridization on solution convergence, reliability, and CPU efficiency. Our results show that hybrid modeling improves surrogate robustness and reduces solution variability with fewer samples, though it increases optimization costs. Additionally, adaptive sampling methods are more efficient and consistent than fixed-sampling surrogate strategies, even across different sampling and dimensionality scenarios.

## 1 Introduction

Chemical process simulators have been widely used in the chemical industry for detailed modeling and analysis. Known as High-fidelity (HF) simulations, they are extensively utilized to support decision-making in complex, dynamic, and stochastic environments.^1−8^ However, they are computationally expensive to evaluate, and simulation-embedded, globally convergent optimization is challenging due to a lack of analytical equations and derivatives. Classical optimization techniques rely on derivative information, and hence they cannot be used directly when derivative information is not available.^9−12^ When optimization speed is constrained by the number of function evaluations (which is often the case with computationally expensive simulations), using finite differences to approximate first-order and second-order derivatives scales poorly with increasing dimensions and may result in low accuracy. As the complexity of simulations grows, the ability to directly optimize systems using commercially available deterministic optimization solvers also becomes a challenge.^13,14^ Moreover, even when algebraic systems of equations representing first-principle high-fidelity models can be developed, it is frequently impractical to directly embed them within largescale optimization formulations, which may lead to intractable formulations. Balancing model accuracy and computational feasibility is crucial when incorporating HF models into extensive optimization formulations.^15,16^ To address the aforementioned challenges, researchers and practitioners have employed data-driven and/or surrogate-based optimization techniques. These techniques are broadly classified as sampling-based and surrogate-based methods. A large body of literature that can be found dating back to 1960s^17−21^ comprising of algorithms that cleverly use samples^4,19−25^ and/or surrogate approximations^6,7,11,26−28^ of the underlying simulation to locate the optima.

With recent advances in machine learning (ML), surrogate-based optimization techniques have attracted a lot of attention, especially for cases where HF simulations are computationally expensive. Various surrogate model choices, such as regression trees, support vector regression, Gaussian-process models, neural networks, and others, have been employed to substitute the HF simulation using data collected from the simulation *a priori*.^6,29−33^ These surrogate models are then used as approximations of HF simulations to expedite and/or enable optimization. By developing a cheaper surrogate function which can be represented algebraically, deterministic local and global solvers can be used to identify the surrogate optima, which could be good approximations of the true optima, if the surrogate is accurate. However, several open challenges exist in surrogate-based optimization (i.e., selection of sampling locations and surrogate model type, training of surrogate models and optimization of surrogate-embedded formulations), which create uncertainty and convergence challenges.^13,27,34^ Several researchers have focused on addressing the determination of the appropriate ML model and have developed strategies for the global optimization of ML models.^30,35−41^ In parallel, researchers have developed adaptive sampling methods that use ML models to locate optima.^34,42,43^ Alternatively, to consider the uncertainty in the process models, researchers have also come up with metaheuristic optimization approaches.^44−48^ Overall, predominant emphasis in the literature on surrogate-based optimization has been on adaptive sampling using black-box ML or surrogate models (i.e., representing HF simulations by a purely data-driven surrogate function).^40,49,50^

The effectiveness of surrogate models relies on data availability, but obtaining sufficient crucial data can be costly or impractical. Insufficient data can lead to inaccurate predictions and hinder optimization. Moreover, black-box models fail to incorporate domain specific knowledge, and this limits interpretability and accuracy, especially in regions that were not sampled. To address this, researchers are exploring ways to embed prior or known knowledge into black-box ML models.^51−57^ One such example is physics-informed ML, in which prior mechanistic knowledge is incorporated as constraints into surrogate model formulations, leading to an improvement in generalization performance, interpretability, and scalability of the ML model.^58−66^ While these physics-informed methods have seen success, their performance depends on accurate physical knowledge. However, in many cases, fully understanding the system’s physics or having that information in advance may not be possible, hence another option is to employ hybrid modeling (HM). There are many scenarios of HM, which involve developing a model that is a combination of submodels of different fidelities. For example, a common scenario in modeling physical and biological systems is the reliance on a computationally cheaper but only “approximately” or “partially correct” low-fidelity (LF) model. To improve the accuracy of representations using LF models, multifidelity surrogate models (MFSMs) have been developed to integrate different levels of data/model fidelity, enabling more robust predictions. MFSMs utilize a composite structure to learn from data sets that include a small set of high-fidelity data and a larger set of inexpensive low-fidelity data. By exploiting the relationship between low-fidelity and high-fidelity data, MFSMs refine the predictions of the overall surrogate model.^42,67−70^ Researchers have shown the accuracy of MFSMs and the improvement over the LF model predictions in several works.^68,69,71^ In our recent work, we have also shown the improvement in the performance of adaptive sampling optimization methods with the inclusion of MFSMs.^42^ For the remainder of the paper, we focus exclusively on MFSMs, using the term “hybrid models” interchangeably with MFSMs.

As surrogate-based optimization and hybrid modeling research have progressed concurrently, we believe there are unexplored connections and interesting comparisons between the two. The emergence of tools like Optimization and Machine Learning Toolkit *(OMLT)*(72) and McCormick-based Algorithm for mixed-integer Nonlinear Global Optimization *(MAiNGO)*,^35,36^ which facilitate and streamline the translation of ML models into Python optimization environments like Pyomo,^73^ makes exploring this comparison an intriguing avenue for optimization.

In this paper, we study surrogate-based optimization of continuous black-box functions, min *f*(*x*) ∀ {*x*^*i*^ ∈ ***x**|x*_*lb*_^*i*^ ≤ *x*^*i*^ ≤ *x*_*ub*_^*i*^}, where *x*_*lb*_^*i*^ and *x*_*ub*_^*i*^ are lower and upper bounds on the variable *x*^*i*^. All inputs (*x*^*i*^) are assumed to be continuous and their upper and lower bounds must be assumed prior to sampling. No further assumptions are made with respect to black-box function *f*, while in practice and in this paper *f* is nonlinear and nonconvex. Several key questions motivate the work presented in this paper, such as:

1. What are the differences in optimization efficiency, reliability and cost between: (a) surrogate methods that rely on *a priori* sampling and optimization of a fixed optimal surrogate, versus (b) adaptive sampling and surrogate-guided methods for optimization?
2. How robust are strategies (a) and (b) to data quality and quantity variations, particularly with respect to consistency of locating locally or globally optimal solutions?
3. What are the effects of surrogate model hybridization within strategies (a) and (b) with respect to solution variability, robustness, and convergence toward a local or global solution?

The objective of this study is to address these three questions through comprehensive analysis and comparison. First, we present a detailed comparison between the surrogate-based and sampling-based optimization methods and show the effect of variability in the data, amount of sampling and dimensionality on the optimal solution. Under surrogate-based optimization, we also explore the effect of solver, and the type of formulation used for optimization on the optimum. Subsequently, we integrate hybrid modeling into both these methods and assess improvements in convergence, reliability, sampling, and CPU speed-ups resulting from hybridization. Specifically, for the analysis with HM in this work, we utilize the MFSM structure for model correction with LF models. Finally, we apply and compare these methods for the optimization of two simulation-based optimization chemical engineering case studies involving extractive distillation and temperature vacuum swing adsorption.

The structure of the article is as follows: In Section 2, we briefly present different types of formulations for surrogate-based optimization with *a priori* sampling such as reduced-space, full-space and ReLU formulations utilizing *OMLT* and *MAiNGO* tools. Additionally, for the analysis with adaptive sampling-based methods, we briefly introduce the data-driven spatial branch-and-bound (*DDSBB*) algorithm. Finally, we introduce the hybrid structure used in this work, namely the MFSM formulation. In Section 3 we use the *multi-Gauss* and *Rastrigin* test functions for analysis and visualization on the impacts of data-variability, availability, dimensionality and hybridization. We then delve into two engineering case studies motivated by temperature vacuum swing adsorption system for CO_2_ capture and extractive distillation. Finally, we extend our discussion in Section 4 and conclude the work with future directions in Section 5.

## 2 Methods

### 2.1 Surrogate-Based Optimization with *A Priori* Sampling

When employing a surrogate-based optimization approach, a common workflow comprises three steps: (1) Creating a sampling design to gather high-fidelity samples, (2) preprocessing the data as needed, and (3) optimizing or ’training’ the machine learning surrogate model to fit the sampled data. The constructed surrogate model can then be used within traditional equation-based optimization solvers to locate the optimum.

Numerous studies have been conducted to compare the effectiveness of surrogate modeling techniques in specific applications.^6,16,27,29,33,74−77^ Recent progress in automatically selecting surrogate models often involves training multiple surrogates and employing a trial-and-error approach to choose the best surrogate based on specific criteria.^16,26^ Nonetheless, current practices for determining the appropriate surrogate model form still rely on domain-specific expertise.^26,30,37^ Throughout this paper, we primarily employ neural networks (NN) as our surrogate model due to their flexibility in approximating nonlinear functions without prior selection of terms and their universal approximation capability,^78^ making them one of the most commonly used options. However, we want to emphasize that beyond our work, many other surrogate or ML models can be used if trained and validated for a particular data set, and are found to be better and/or simpler approximators. In fact, we believe that some of the findings of this work would still hold, regardless of the surrogate model selection.

Choosing the right quantity of sample points and the method for generating those samples is a crucial stage in building a surrogate model. Generally, a greater number of sample points provides more insight into the underlying model being approximated, albeit at a higher computational cost.^79,80^ Earlier research has explored the impacts of sample size and sampling method on various surrogate modeling techniques.^15,81^ The findings from these studies highlight that the accuracy of a surrogate model relies on the quantity and distribution of samples employed in its construction.

**Figure 1:**
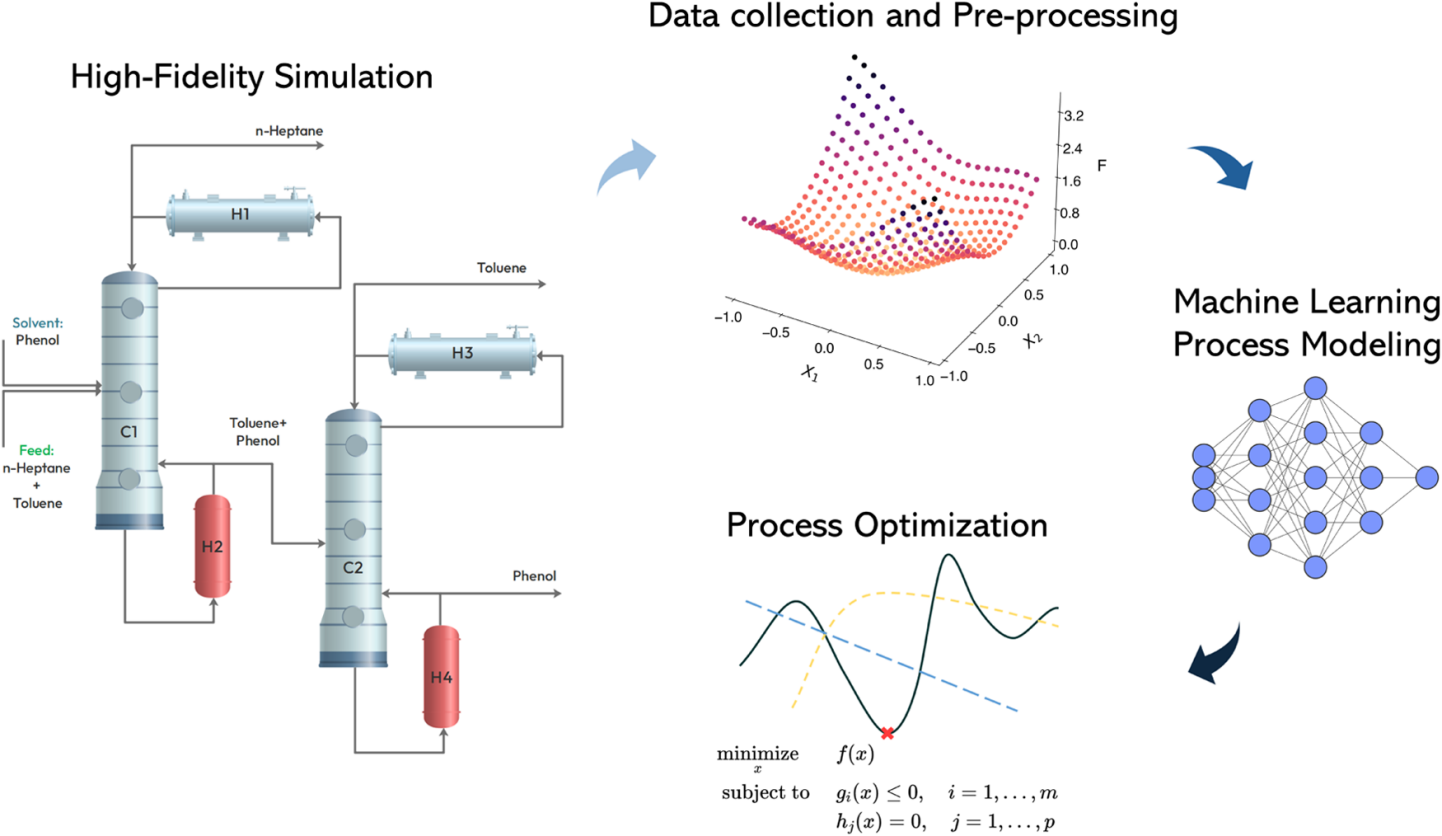
Process of constructing a surrogate model with *a priori* sampling and subsequent optimization.

In some cases, fixed *a priori* sampling is necessary due to limitations in data generation, while in other instances, data can be generated iteratively by running HF simulations as needed. This *a priori* sampling approach for surrogate-based methods is frequently employed in simulation optimization.^16,27,75,81^Figure 1 provides a graphical overview of the surrogate-based optimization workflow with *a priori* sampling. Additionally, there are techniques that use sampling criteria (e.g., expected improvement methods) to iteratively sample and update the surrogate model,^33,80,82,83^ however, the scope of this paper does not encompass these methods.

The surrogate models constructed are then fixed and optimized using traditional equation based solvers. Very recently, several tools^36,72,84,85^ were developed that facilitate formulating ML models into Python optimization environments such as Pyomo.^73^ Specifically for the analysis in this work, we use two such tools *Optimization and Machine Learning Toolkit (OMLT)*,^72^ and *McCormick-based Algorithm for mixed-integer Nonlinear Global Optimization (MAiNGO)*.^36^ Furthermore, there are various ways in which the ML model can be formulated for optimization.^36,72,86,87^ Below, we briefly outline a few of the formulations we employ in this work.

#### 2.1.1 Full Space Formulation

**Figure 2:**
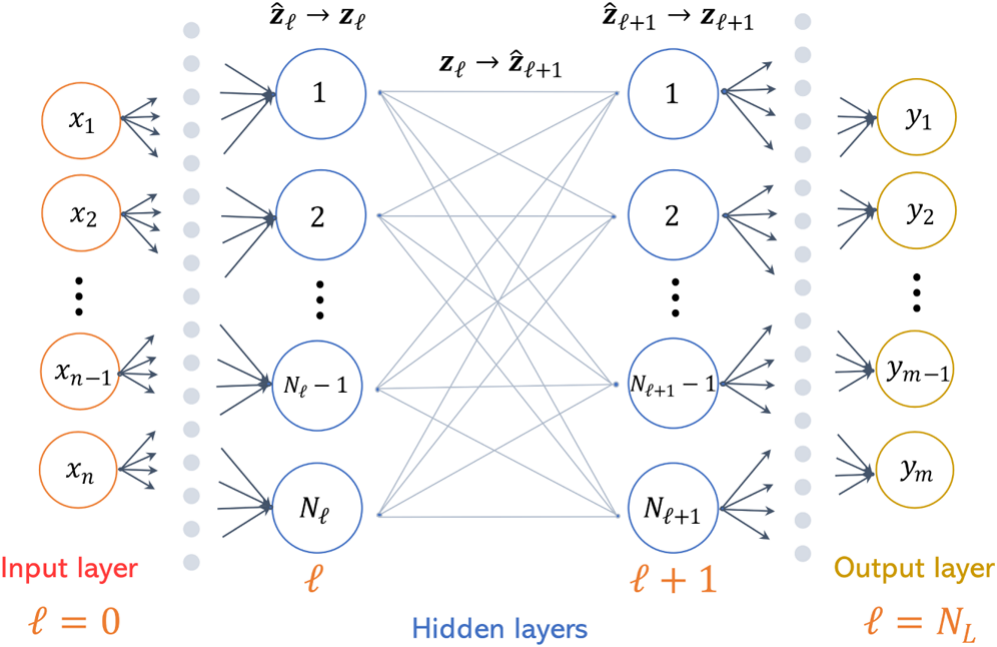
Neural network architecture depiction.

We consider a feed-forward NN as illustrated in Figure 2. Let the input have a dimensionality of *n*, and the output a dimensionality of *m*. The network comprises layers indexed from , where  corresponds to the input layer,  corresponds to the output layer, and  represent the hidden layers. The input vector to any layer *k* is a linear combination of the output of the previous layer, denoted by ***z***. The preactivation and postactivation vectors are indicated as  and , and ***W***, ***b*** represent the weight and bias matrix, respectively. The element-wise operations showcasing the NN representation are provided by eqs 1–4. The vector ***x*** can be denoted as ***z***_0_ to represent the input layer to the NN and to align with the notation.
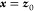
1
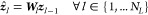
2
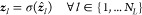
3
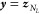
4

Equation 2 represents the preactivation values obtained from the weights, biases, and outputs of the preceding layer, eq 3 incorporates the activation function, and eq 4 links the last layer to the output. For the full space formulation, the intermediate variables associated with the NN representation, and the activation function constraints are formulated explicitly in the optimization problem.

#### 2.1.2 Reduced Space Formulation

The reduced space formulation on the other hand uses one expression to capture the NN variables and constraints. Consider a feed-forward NN architecture as illustrated in Figure 2.
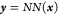
5The input vector ***x*** is mapped to the output vector ***y*** to as ***x*** → ***y*** with the operator *NN*(*) that represents the encoded NN function, that internally uses the activation function, weights and biases associated with it. The activation function constraints can be any smooth function of choice such as tanh, sigmoid, soft-plus etc. The intermediate neural network variables and activation functions are hidden from the underlying optimizer and the neural network is represented by one constraint shown in eq 5. This results in an optimization problem with lower complexity in comparison to the full space formulation.

#### 2.1.3 Mixed-Integer Linear Formulation Using ReLU Activation Functions

The ReLU activation function is defined as *z*_*i*_ = *max*(0, *ẑ*_*i*_). While it is possible to formulate ReLU in both full-space and reduced-space representations, the constraints that arise are not smooth. This can be handled by representing ReLU NNs using *big-M* constraints. The variable *q*_*l,i*_ represents a binary indicator that decides whether the output from node *i* on layer *l* is 0 or takes the value *z*_*l,i*_. The constants *M*_*l,i*_^*U*^ and *M*_*l,i*_^*L*^ are large constants employed to enforce the ReLU logic.
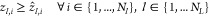
6
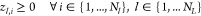
7
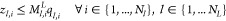
8
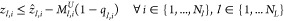
9To assess the ReLU formulation for optimization, we employ OMLT.^72^ Instead of manually selecting arbitrary large values for the *big-M* constants, OMLT automatically determines these values by propagating the bounds on the input variables.

### 2.2 Multifidelity Surrogate Models

Multifidelity surrogate models (MFSMs) are hybrid models that integrate multiple single-fidelity models using weighted coefficients. By combining models of varying fidelities, MFSMs capitalize on the unique strengths of each individual model.^51,67−71^ The most common configuration for MFSMs involves data sources of varying fidelity levels. HF samples are typically scarce, due to their computational or experimental costs. Nevertheless, HF data provides highly accurate and reliable insights, representing the most precise understanding of the system at hand. In contrast, LF data is generally inexpensive to obtain but may lack the same level of accuracy. LF data often captures the primary trends of the underlying system, exhibiting correlation with HF data. One such example is a computational fluid dynamics (CFD) model evaluated on a fine grid (HF model) versus lower fidelity versions of the CFD model, such as a ML surrogate or the same CFD model evaluated on a coarse grid (LF model).^69,88−90^ In other instances, such as kinetic reaction systems or biological systems and pathways, LF models may be built by omitting certain terms due to incomplete information of the system.^91−94^ A key challenge in MFSM model development lies in effectively combining HF and LF information to leverage the high accuracy of HF models and the computational efficiency of LF models to the fullest extent. A widely used correlation^42,67−71^ to build MFSMs is given in eq 10, where ***y***_*L*_, ***y***_*H*_ represent the low-fidelity and high-fidelity data respectively, ρ(***x***) is the multiplicative correlation surrogate and δ(***x***) is the additive surrogate. In a more general way, we can rewrite it as a function of the input ***x*** and the LF data as shown in eq 12.
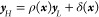
10
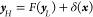
11
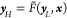
12For the cases where there is no LF model that pre-exists, we can build a MFSM model by utilizing a combination of surrogate models. Although any different combination of surrogates can be used, in our work we utilize the pseudocode shown in Algorithm 1 for formulating the MFSMs (when a LF model does not exist). Here, ***y***_*LF*_, ***y***_LF_^*^ represent output from a support vector regression model (SVR) and the NN, respectively. *N*_LF_, *N*_HF_ is the number of low fidelity training data points and total HF data points. β_1_, β_2_ represent the regularization weights and ϕ_SVR_, ϕ_NN_ represent the associated parameters with SVR and NN respectively. While various surrogate models can serve as the LF model, SVR is chosen for its relatively lower complexity compared to other options. We also tested other surrogate model candidates for the case studies used in this paper and a detailed analysis and discussion on is included in Section 7 of the Supporting Information. As illustrated in Algorithm 1, if a LF model already exists, we omit constructing an LF model using SVR. Conversely, for cases lacking a pre-existing LF model, we develop one employing SVR. The selection of a surrogate model for building a LF model depends on the problem’s complexity. We utilize a LF model that is sufficiently simple, yet capable of capturing HF data trends, with any residual errors addressed in the subsequent step. Throughout the remainder of this document, we once again emphasize that the term “hybrid models” specifically refers to and utilizes MFSMs.
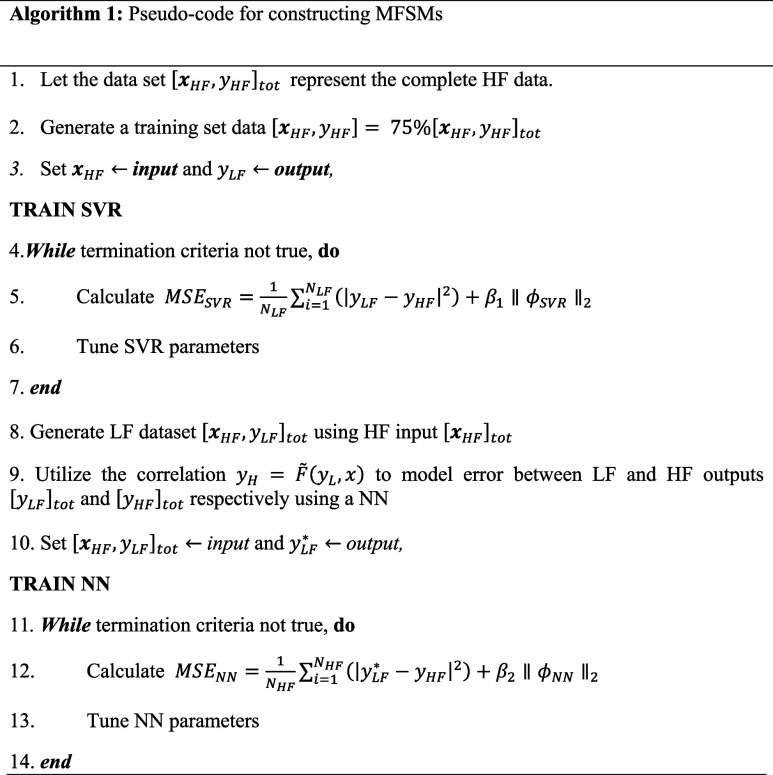


### 2.3 Adaptive Sampling-Based Optimization

Another class of methods for simulation optimization is adaptive sampling methods. Unlike fixed *a priori* sampling, these methods start with a smaller set of samples in the search space and then adaptively determine where to sample next using sampling criteria or heuristics. Some methods rely solely on sample data,^4,11,20−23^ while others employ surrogates that are adaptively refined during optimization.^82,95−99^ Often, there is no universal surrogate that represents the whole space and capitalizing on that, a few methods use a collection of local surrogate models around each evaluated point.^41,100^ For the purpose of comparing fixed *a priori* sampling with adaptive sampling methods, we select an adaptive method that we have previously developed in-house, namely the data-driven spatial branch-and-bound (DDSBB) algorithm. DDSBB incorporates adaptive sampling and integrates surrogate modeling for black-box optimization. We demonstrated the algorithm performance by comparing it with existing solvers,^42,43^ showcasing its ability to provide consistent solutions and solution lower bounds. For all the subsequent analysis in the paper involving adaptive-sampling methods, DDSBB is employed. In the following paragraph, we provide a brief introduction to DDSBB.

#### 2.3.1 Data-Driven Spatial Branch-and-Bound—DDSBB

The DDSBB algorithm is a data-driven equivalent of the spatial branch-and-bound algorithm. The algorithm employs a linear programming formulation to create convex underestimators of collected data, which in case of simulation optimization are HF samples. These underestimators, being convex, serve as relaxations and can be optimized globally using efficient local solvers. DDSBB utilizes samples from a HF simulation as an upper bound (UB) and the minimum of the convex underestimator as the lower bound (LB). The search space is partitioned through branching, node selection, and pruning rules, with adaptive sampling in the nonpruned subspaces.

**Figure 3:**
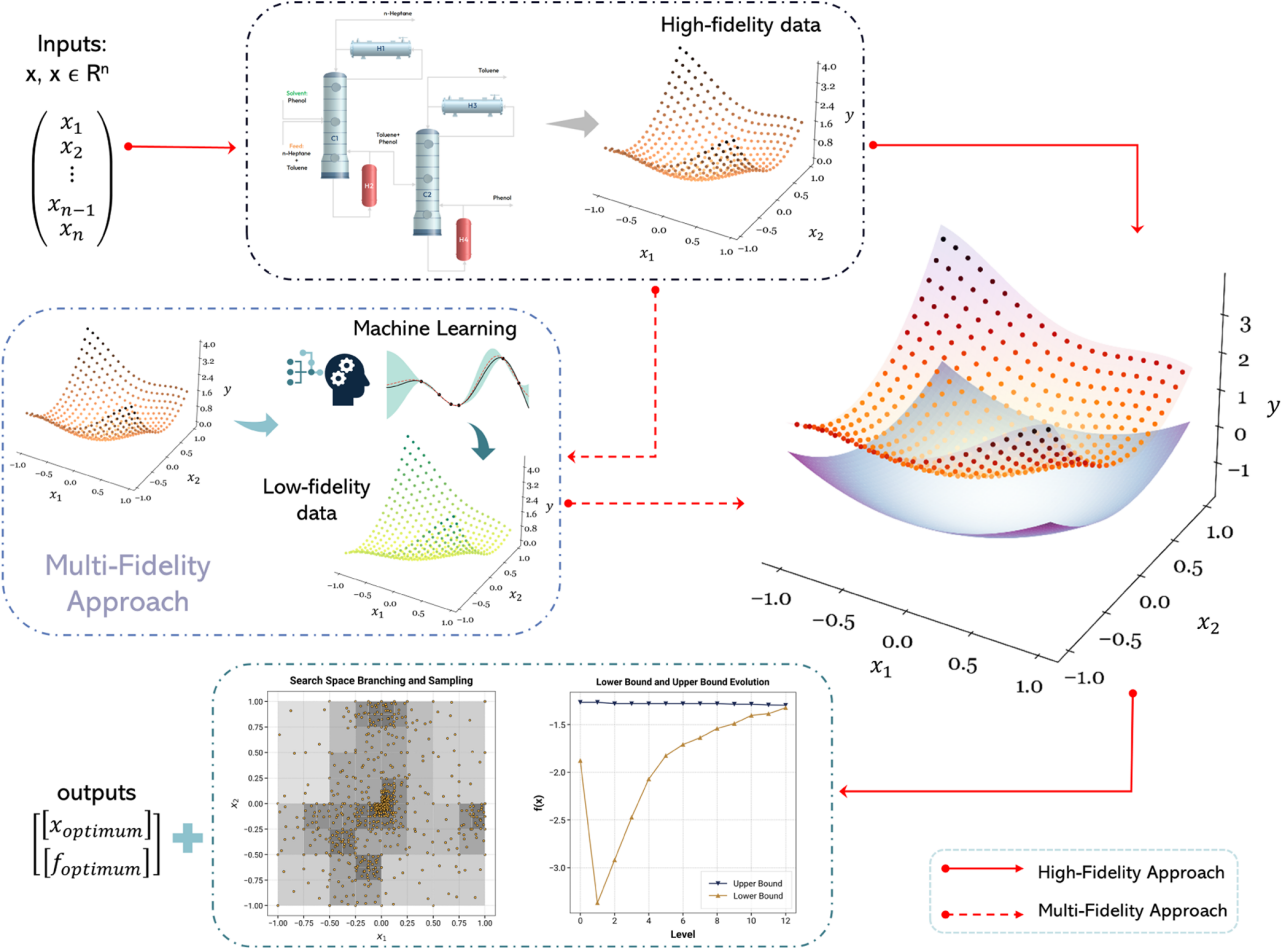
Overview of the DDSBB algorithm and its high-fidelity and multifidelity approaches.

Additionally, the algorithm leverages a surrogate modeling technique to develop LF ML surrogates of HF data. These LF models facilitate the generation of LF data, which, when combined with HF data, improve the validity of convex relaxations during the branch-and-bound process. We refer to this strategy as the “multifidelity” (MF) approach. Our analysis with benchmark problems in previous work demonstrated an improvement in the algorithm’s performance when employing this approach. Throughout the remainder of this paper, we employ SVR models as the LF surrogate under the MF-DDSBB framework. In Figure 3, we show the outline of the DDSBB algorithm.

Among the three methods tested using DDSBB, HF-DDSBB employs exclusively HF samples obtained from the underlying model for the optimization process. MF-DDSBB adopts an alternative approach by first utilizing the HF samples to develop an LF-SVR model. This LF-SVR model is then used to generate additional LF samples, which, together with the original HF samples, are incorporated into the optimization process. It is important to note that the MF approach of this algorithm is not the same as the MFSM approach studied in this article, since it is simply underestimating data from HF and LF simulations but does not build composite models nor does it explore correlations between the two. HM-DDSBB is utilized when hybrid modeling is necessary. If the LF model is efficient but slightly inaccurate, the MFSM approach is employed to correct the LF model and generate additional data, termed LF* samples. These LF* samples are designed to match the accuracy of the HF data. The combined HF and LF* samples are subsequently used in the optimization process.

## 3 Results

### 3.1 Two-Dimensional Case Study—The Multi-Gauss Function

**Figure 4:**
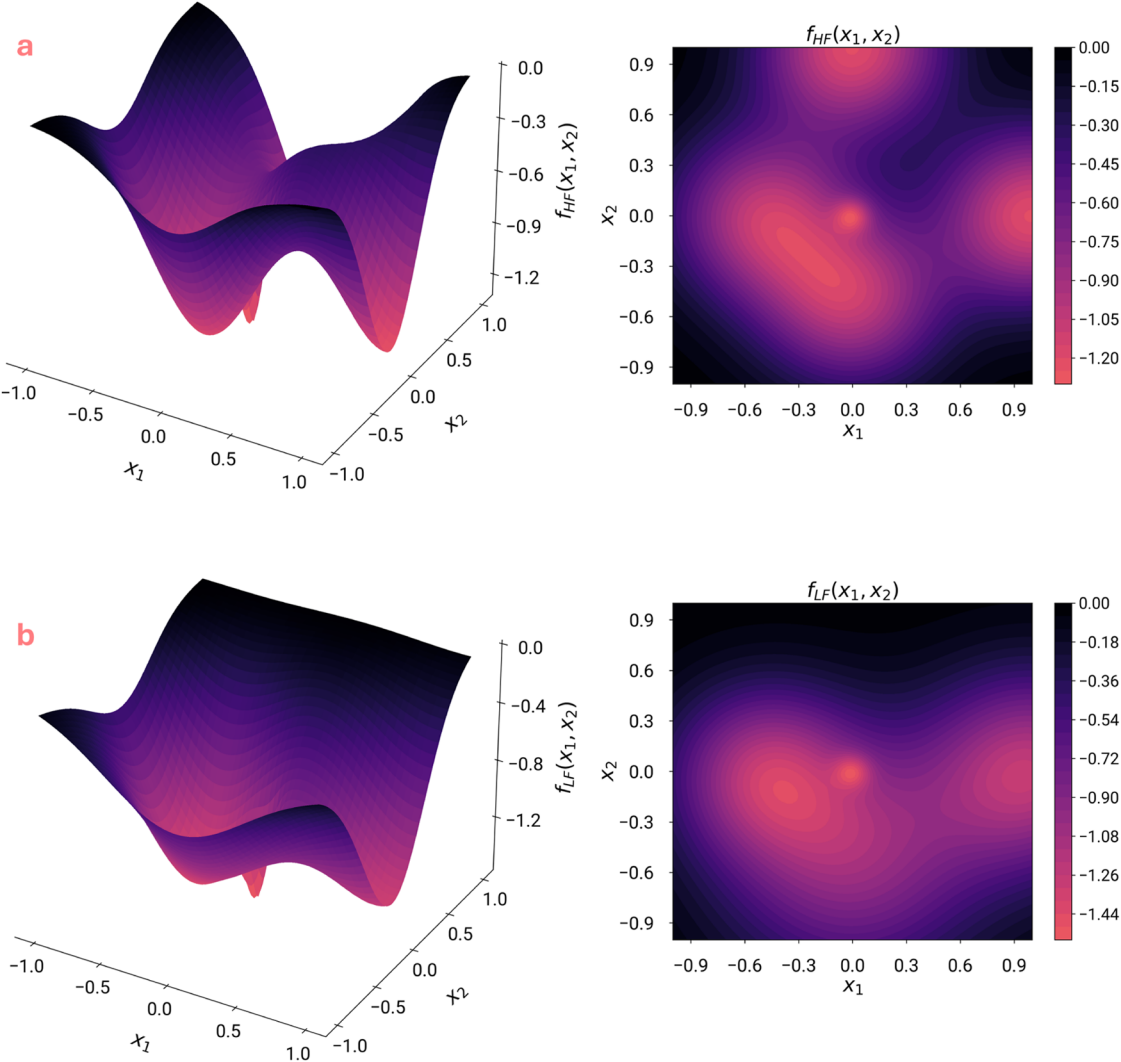
Surface and contour plots of (a) *Multi-Gauss* and (b) *Low fidelity Multi-Gauss* functions.

To visualize the analysis in the subsequent sections, we utilize the two-dimensional *Multi-Gauss* test function.^101^ This function is treated as a black-box simulation and optimized with the methods outlined in Section 2. The function is continuous and has two input variables, *x*_1_ and *x*_2_, both within the range of [−1, 1]. The profile is shown below in Figure 4a, and it has a global minimum value of *f*_HF_^*^ = −1.296954 located at [*x*_1_, *x*_2_] = [−0.01354, −0.01354]. The function form connecting the two variables *x*_1_ and *x*_2_, is shown below.
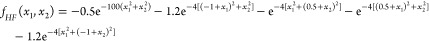
13Additionally, we generate a low-fidelity variant (shown in eq 14) of this benchmark by eliminating certain terms from the high-fidelity functional form (eq 13). This LF function is then employed in the analysis using hybrid modeling. The function involves two input variables, denoted as *x*_1_ and *x*_2_, both confined to the range of [−1, 1]. The LF function’s profile is shown below in Figure 4b, and it has a global minimum value of *f*_LF_^*^ = −1.547785 located at [*x*_1_, *x*_2_] = [−0.0117, −0.01204]
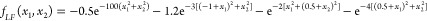
14

To construct the surrogates for optimization, we generate the input–output pairs to create training and validation data sets using Latin hypercube sampling (LHS).^102^ LHS generates space filling designs that are uniformly distributed across the domain and can be tailored for any desired sample size. We use NNs as surrogates, trained and optimized using TensorFlow.^103^ For the NN architecture, we use a feed forward network with one input layer, two hidden and one output layer respectively with tanh activations. Each hidden layer consists of 100 nodes and the network is trained with 500 samples for 4000 epochs. In the case of the ReLU formulation, we use the same NN architecture. This configuration was determined through hyperparameter tuning using Bayesian optimizer with Keras Tuner^104^ and required an additional ∼1500 s for tuning. The NN architecture with R^2^ scores greater than equal to 0.99 and the least mean square error was then chosen as the optimal configuration. This was done to ensure that we identify the best surrogate model based on the data that we have collected *a priori*.

**Figure 5:**
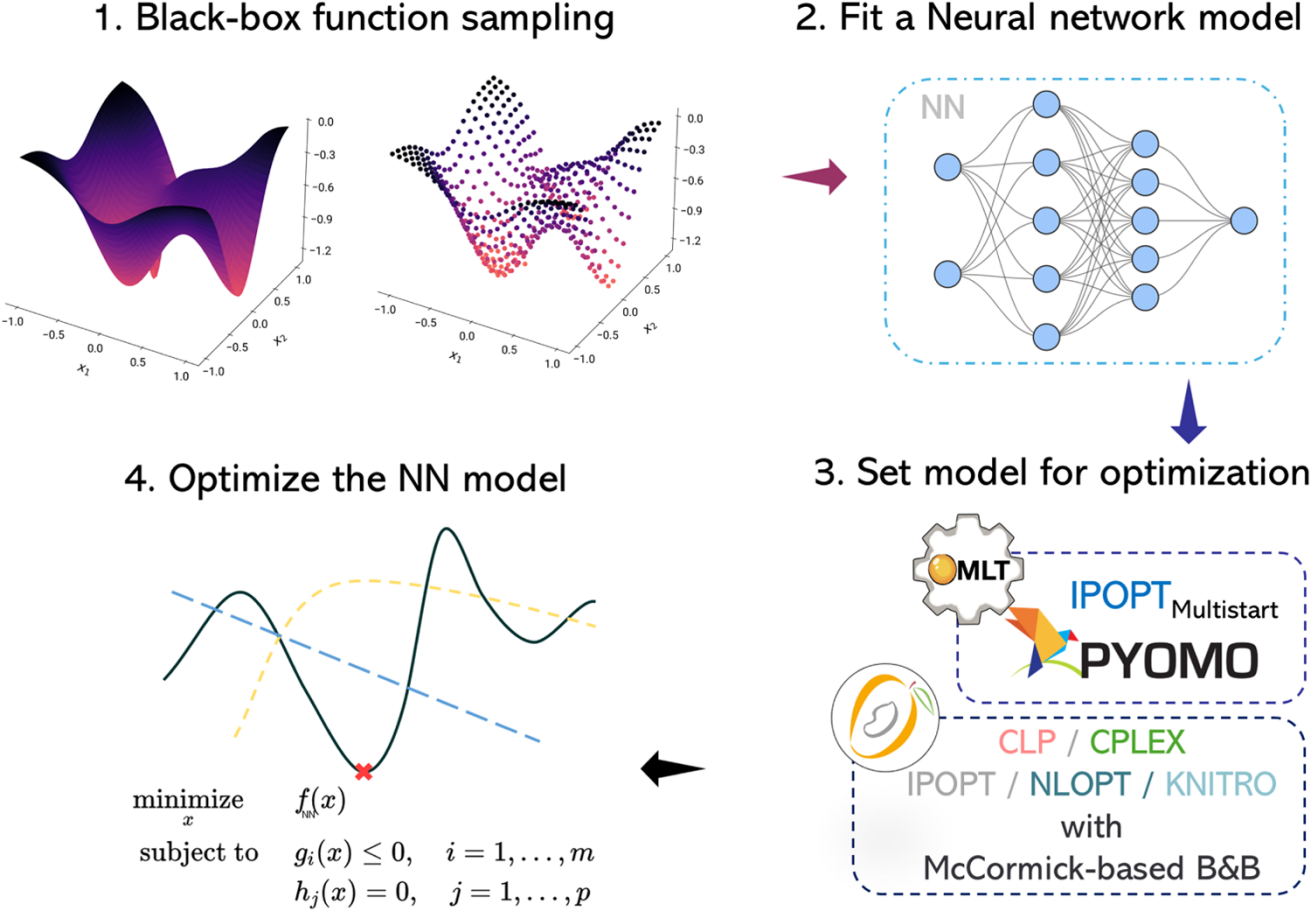
Process of fitting a surrogate and optimization for the *a priori* sampling based surrogate optimization.

For the *a priori* sampling-based surrogate model optimization, we take the following approach for constructing the surrogate model and the subsequent optimization. In step 1, we generate a LHS design with ‘n’ samples that fills the domain of interest uniformly. In step 2, we use these samples for training and cross-validation of the NN model with the architecture identified from the hyper-parameter tuning, by minimizing the mean squared error (MSE). The trained NN model is then embedded into an optimization problem using the tools *OMLT* and *MAiNGO* in step 3. Finally, in step 4, we use the available optimization solvers within each environment to find the optimum. The outline of this process is shown in Figure 5. We utilize the reduced and the full-space, MILP ReLU formulations with *OMLT* and reduced space formulation with *MAiNGO*. To increase the probability of identifying the global surrogate optimum when using OMLT, we use the multistart IPOPT^105^ solver for the reduced and full space formulations, and Gurobi^106^ or Baron (version 24.5.8)^107^ solvers for the ReLU formulation. *MAiNGO* on the other hand, has its own tailored global optimization algorithm, which we employ here.^36^ We further emphasize that our goal is not to compare the tools *OMLT* and *MAiNGO*, but to use them for formulating and solving optimization problems with embedded surrogates. This allows us to utilize equation-based solvers to analyze how differences in sample size and variability, affect variation in the global solutions obtained.

We use DDSBB algorithm for adaptive sampling methods and evaluate both the HF and MF approaches outlined in Section 2.3.1. In the HF approach, there is no fitting of ML models or direct optimization of the ML model or the underlying function.

#### 3.1.1 Effect of Sampling Reinitialization on the Variability of Optimum Solution

To assess the impact of variations in the sampling design on the surrogate model and the variability of the optimal solution in the subsequent step, we iterate through the process outlined in Figure 5 30 times. Throughout each repetition, all steps within the loop remain consistent, except for step 1. In each run of the 30 repetitions, the sampling design undergoes a modification with random initialization based on LHS design, while maintaining a constant number of samples. The NN architecture remains unchanged, but it is retrained using the updated sampling design to minimize fitting errors and identify the best fit. This iterative approach allows us to investigate whether changes in the sampling design lead to variability in the optimal solution. For DDSBB, we use a sampling limit of 500 samples for a fair comparison with *a priori* sampling. The absolute and the relative tolerance values for the upper and lower bound gaps are set to 0.05 and 0.01 respectively. Similar to the *a priori* sampling methods, the optimization process was repeated 30 times, each time with a different sampling initialization to check the variability in the solution.

**Figure 6:**
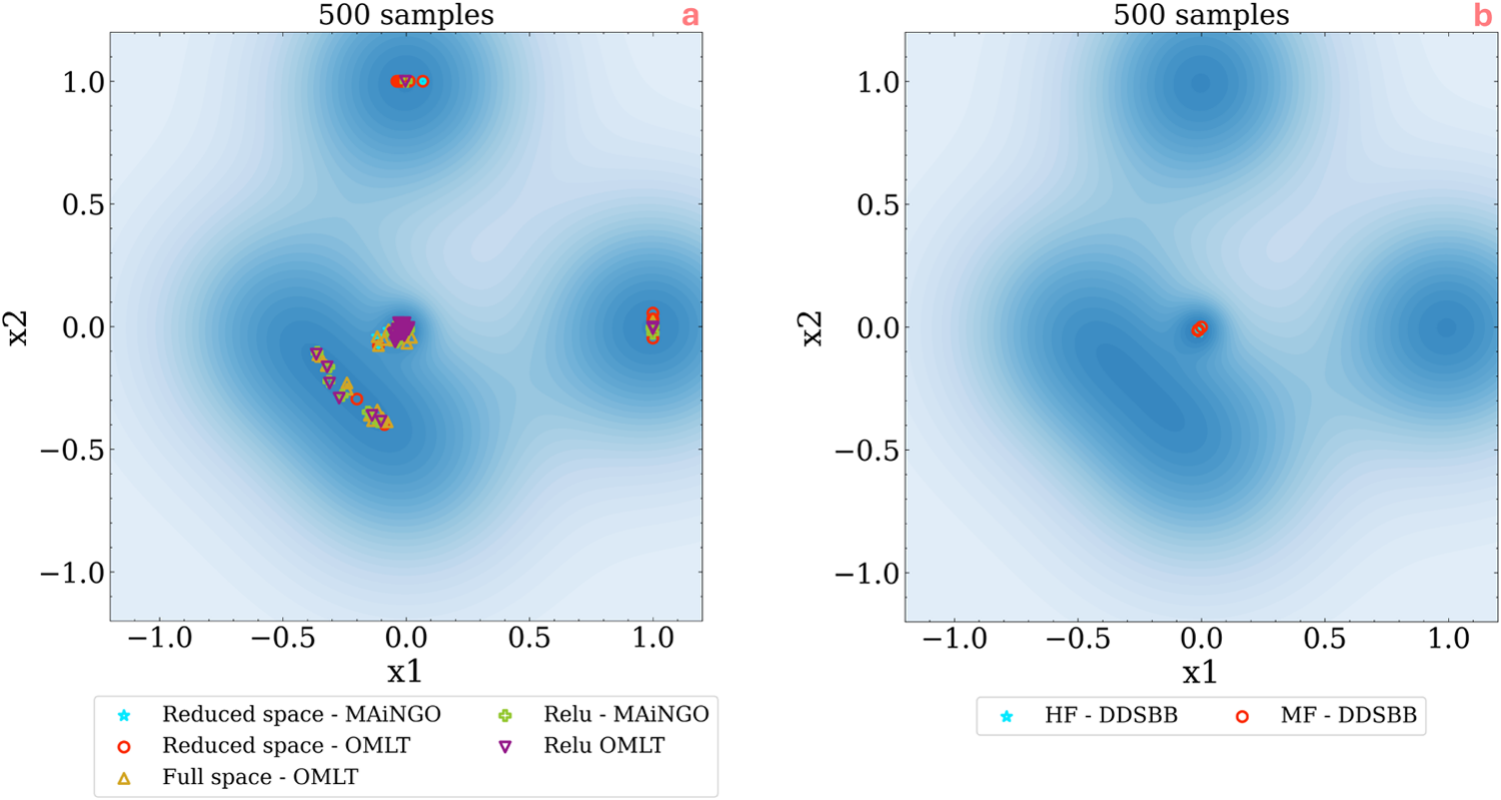
Visualizing the effect of sampling reinitialization on the variability of optimum solution for (a) *a priori* sampled surrogate based optimization vs (b) adaptive-sampling based optimization.

Figure 6a illustrates the results on solution variability with sampling reinitialization. It is evident that there is variability in the solution obtained through *a priori* sampled surrogate methods across all three optimization formulations. This variability arises due to the data-dependent parameters of the NN model. Changes in the sampling design for each run in the 30 repetitions lead to alterations in the associated model parameters during the training step. Consequently, this influences the optimization formulation, ultimately resulting in variability in the solutions.

In contrast, when employing adaptive sampling methods like DDSBB, minimal to no variation in the solution is observed in Figure 6b. This consistency holds true for both the HF and MF approaches. For a given number of HF samples, MF-DDSBB generates additional data to be used in optimization. This additional data enhances the algorithm’s ability to construct underestimators more effectively, even in regions with sparse HF samples, which would not be possible using HF data alone. Consequently, in the case of nonlinear functions, this approach enables the algorithm to locate the optimum more accurately. We have demonstrated the effectiveness of this methodology in one of our previous works.^108^ The errors in objective function values and time statistics corresponding to each method tested for the 30 repetitions are shown in Table 1.

**Table 1:** Objective Error and Time Statistics Comparison for *a Priori* Sampling Versus Adaptive Sampling Approaches

tool	maximum time (s)	minimum time (s)	average time (s)	maximum error (%)	minimum error (%)	average error (%)
reduced space - MAiNGO	147.1	100.1	118.1	15.9	0.1	6.8
reduced space - OMLT	145.6	97.7	115.9	15.4	6.2	7.4
full space - OMLT	149.9	101.2	119.8	15.9	0.1	5.8
Relu - MAiNGO	380.7	198.2	325.2	7.3	0.1	3.9
Relu OMLT	194.2	128.6	163.4	7.7	0.1	4.1
HF - DDSBB	5.5	1.4	3.6	1.3	0.0	0.6
MF - DDSBB	7.5	1.4	4.9	1.3	0.0	0.1

#### 3.1.2 Effect of Sampling Number on the Variability of Optimum Solution

**Figure 7:**
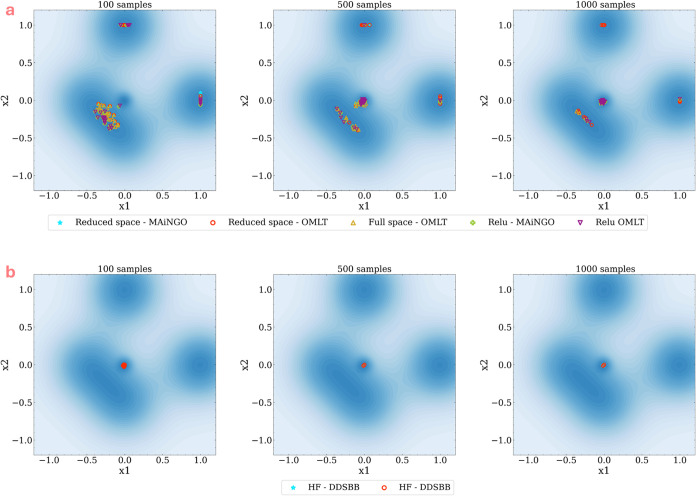
Visualizing the effect of sampling number with reinitialization on the variability of optimum solution for (a) *a priori* samples surrogate based optimization (top panel) vs (b) adaptive-sampling based optimization (bottom panel).

To investigate the impact of the number of samples in the sampling design on solution variability, we repeat the aforementioned analysis with three different sample sizes: 100, 500, and 1000 samples. Employing the same NN architecture as in the previous analysis, we iterate through the optimization process 30 times for each sample number. Figure 7 presents the results depicting the influence of increasing sample sizes on solution variability.

For surrogate-based methods with *a priori* sampling, variability is still observed with 1000 samples, although it decreases as the sample number increases from 100 to 1000 samples. The function profile is captured more effectively with a larger number of samples, and this reduces the impact of variation in the sampling design on the model parameters. Consequently, when the surrogate model is trained, the associated variability in the model parameters diminishes, leading to reduced variability in the solution.

In the adaptive sampling approach with DDSBB, we maintain the same algorithm parameters while altering the sampling limit to three different sample sizes (100, 500, and 1000 samples). Similar to the previous results, minimal variability in the solution is observed for both HF and MF cases. This variability further decreases as the sample number increases, demonstrating the robustness of the adaptive sampling technique across varying sample sizes. Among the HF and MF approaches, the average/mean of the solution variability is relatively lower for the MF approach, consistently across the 3 sample sizes tested. As mentioned before, this is because under the MF approach, in addition to the HF samples from the underlying function, additional samples from the LF-SVR model are used in the optimization process. This additional information helps the algorithm in locating the global solution more accurately.

#### 3.1.3 Effect of Hybrid Modeling on Solution Variability

In most practical applications, it is common to have an LF model that is less accurate but significantly less computationally demanding than the HF simulation. In order to assess if there is any reduction in variability of the solution, we repeat the analysis from above, but this time utilize a multifidelity hybrid model across all methods. Under the surrogate-based methods with *a priori* sampling, the LF multi-Gauss function, along with the HF function, is employed to construct a MFSM hybrid model. We follow the same optimization pathway and repetitions shown in Figure 5, replacing the surrogate model with the formulated MFSM.

**Figure 8:**
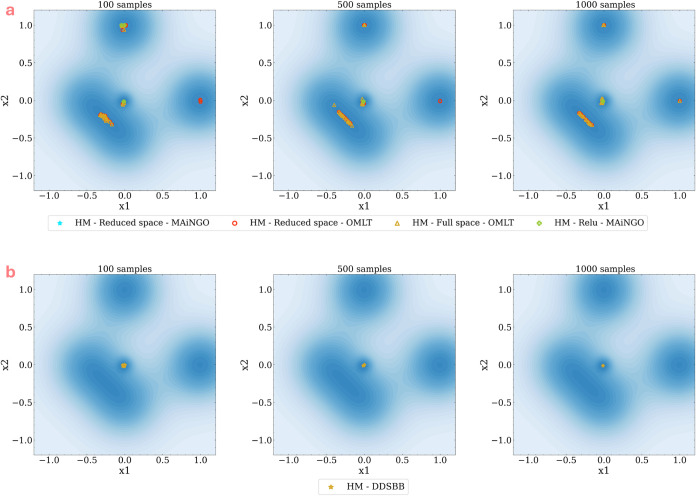
Visualizing the effect of sampling number with reinitialization on the variability of optimum solution with hybrid modeling, for (a) *a priori* samples surrogate based optimization (top panel) vs (b) adaptive-sampling based optimization (bottom panel).

Figure 8a presents the results with respect to the sampling number and reinitialization on the variability of the solution with hybrid modeling. It is evident that there is still variability in the optimum solution discovered, albeit less compared to the variability observed with the black-box surrogates in Figure 7. It should be noted that the number of samples used in this experiment remains the same as in the previous analysis. This reduction in variability is attributed to the MFSM structure, which leverages the predictions from the LF model in addition to the HF values, thereby enhancing the accuracy and robustness of the model. Consequently, the variability in the solution decreases. Consistent with the analysis in Figure 7, a similar trend is observed in Figure 8: the variability decreases as the number of samples in the sampling design increases.

For the adaptive sampling approach with DDSBB, we employ the pseudocode from Algorithm 1. Similar to the earlier analysis, we keep the algorithm parameters constant but vary the sampling limit across three different sample sizes (100, 500, and 1000 samples) while enabling the MFSM approach. Figure 8b illustrates the results obtained through adaptive sampling optimization. Once again, the variability in the solution is minimal when compared to *a priori* sampled surrogate optimization even in the case of 100 samples. Furthermore, this variability continues to decrease as the sample number increases.

#### 3.1.4 Visualizing the Relative Error and Computational Requirements with Sampling

**Figure 9:**
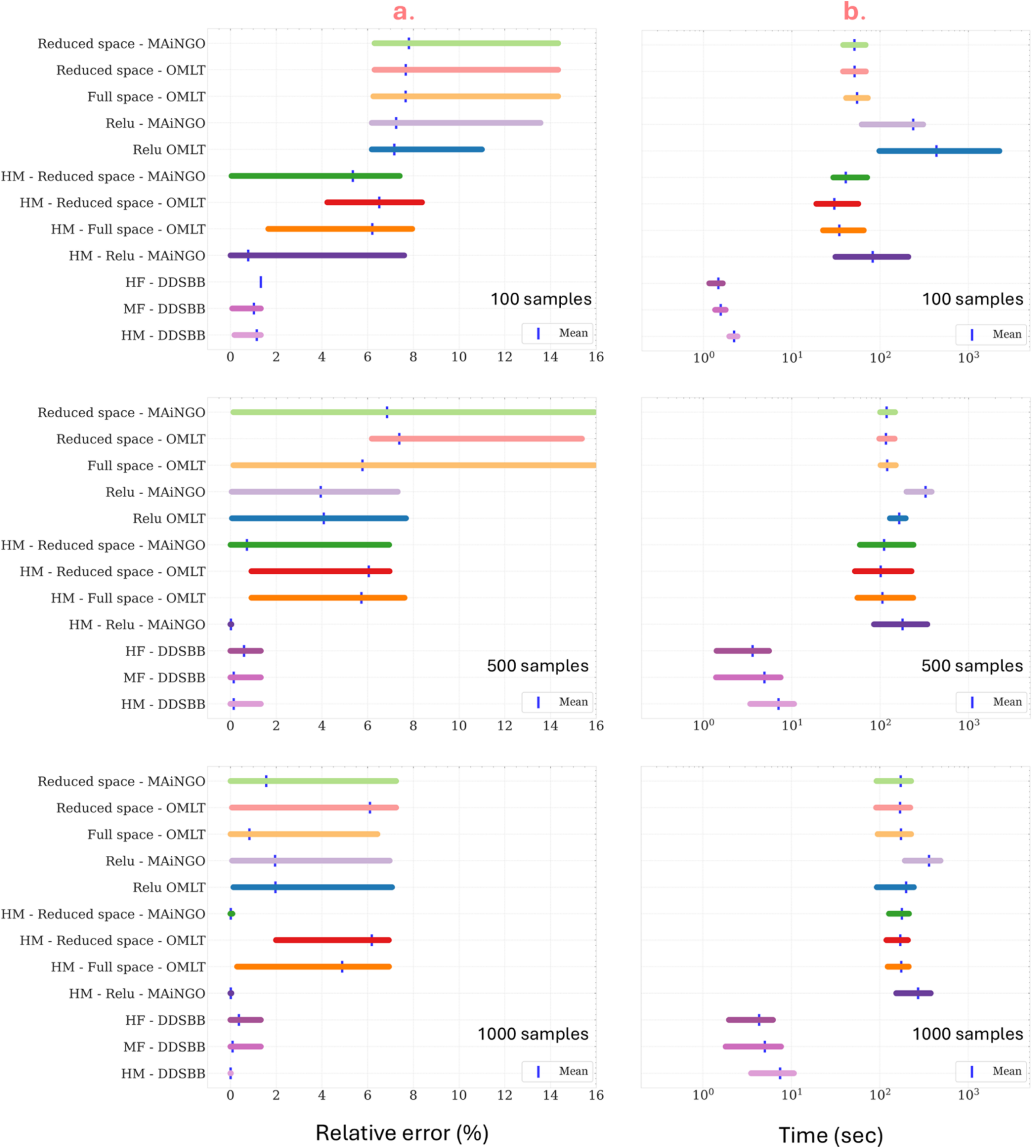
(a) The relative error of the discovered solution with respect to the true optimum. (b) The time requirements for modeling and optimization, for *a priori* samples surrogate-based optimization and adaptive-sampling-based optimization with black-box and HM methods.

For a more quantitative understanding of solution variability, we present relative error plots and CPU requirement plots for both *a priori* sampled surrogate optimization and adaptive sampling-based optimization, with and without hybrid modeling. The *x*-axis of the plot in Figure 9a depicts the relative error of the solution obtained with respect to the true optimum, while in Figure 9b, the *x*-axis represents the CPU requirements for optimization. Consistent with previous findings, the relative error is reduced as the number of samples increases. It is also evident from Figure 9 that the relative error obtained under hybrid modeling methods is less than their counterpart black-box methods.

Among the surrogate-based methods compared, it can be observed that the ReLU MIP formulation for black-box surrogates yields the lowest error percentage relative to the true solution. Since solving the formulation under MIP guarantees a global solution, the quality of the solution depends solely on the model’s fit and accuracy. It is important to note that the same NN architecture is used for all three sample sizes analyzed. However, because the final model structure depends on the sampled data, the same architecture may sometimes lead to overfitting or underfitting. Overfitting, in particular, can result in a more complex optimization formulation and, consequently, longer solving times, especially when sample sizes are small, and the model is tailored to fit this data.

For the reduced and full-space formulations using OMLT, IPOPT is employed to locate the optimum. Although multistart is enabled to increase the chances of finding the global solution, it may still converge to local solutions. The solution variability is relatively lower in the full-space formulation compared to the reduced-space formulation. Since the entire network structure is used as constraints in the full-space formulation, it provides the solver with more information to locate the solution, although this leads to a more complex problem.

The robustness of the model improves with the inclusion of HM, which consequently leads to lower variability in the solution when optimized. Specifically, the accuracy of the model and the smoothness of the model improves when HMs are used. As a result, the solver is better able to avoid getting trapped in local minima. In contrast, black-box methods, which can produce noisier profiles, increase the likelihood of the solver converging to local solutions. A more detailed analysis on this is shown in Supporting Information – Section 8, where we show the MFSM fit and the plot its derivative profiles. However, it is important to note that the LF model can be nonlinear, as observed in this case, leading to a more challenging MINLP formulation with ReLU structures, requiring an MINLP solver to find the solution, thereby increasing computational time requirements. A detailed analysis of the MINLP formulations is provided in Supporting Information – Section 2. For the reduced and full-space formulations with HM, the problem remains an NLP, and a similar trend to that of black-box surrogates is observed. With the adaptive sampling methods, the underlying function is optimized indirectly and by adaptively constructing underestimators, the above challenges can be avoided. This contributes to the consistency in the optimum solution despite changes in the sampling design and even with HM.

Figure 9b showcases the time requirements for training the model and optimization. Notably, the time requirements increase with an increase in the number of samples. This increase is attributed to the longer training times for the NN model as the sampling size grows. Additionally, it is noteworthy that the time requirements for adaptive sampling optimization with DDSBB consistently remain at least an order of magnitude lower when compared to surrogate optimization with *a priori* sampling. This indicates the efficiency and computational advantage of the adaptive sampling optimization approach over the surrogate-based method with *a priori* sampling.

With HM, the time required to train the model and optimize it,increases with an increase in the number of samples. Furthermore, when compared to the black-box methods, the time requirements for the HM methods are higher. This can be attributed to the more complex composite structure of the MFSM, resulting in higher time requirements. Overall, from Figure 9, we observe that HM helps in reducing the variability in the solution, even with a smaller number of samples, but simultaneously results in higher time requirements to train and optimize the models. This approach is advantageous when leveraging LF models to reduce sampling costs from HF simulations.

### 3.2 Effect of Dimensionality—The Rastrigin Function

To assess the effects of dimensionality, we utilize the *Rastrigin function*, with a functional form that can be generalized to *n* dimensions. Similar to the above analysis, this function is treated as a black-box simulation and optimized with the methods outlined in Section 2. The function is continuous and has input variables, *x*_*i*_, *i* = 1, 2,..., *n*, all within the range of [−1, 1]. It has a global minimum value of *f*_*HF*2_^*^ = 0 located at [*x*_*i*_]_nx1_ = [0]_nx1_. The function form connecting the two variables *x*_1_ and *x*_2_, is shown below.
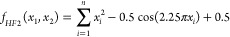
15We also generate a low-fidelity variant of this benchmark by eliminating certain terms from the high-fidelity functional form. It has a global minimum value of *f*_*LF*2_^*^ = 0 located at [*x*_1_, *x*_2_] = [0, 0]. The function form connecting the two variables *x*_1_ and *x*_2_, is shown below.
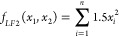
16Similar to the previous analysis, to evaluate the effect of sampling variation on the surrogate model and optimal solution variability, we repeat the process in Figure 5 30 times, modifying only the sampling design in step 1. The NN architecture remains the same across the repetitions under each dimensionality considered. The NN configurations with tanh and ReLU activations that have the lowest training and validation errors were identified based on hyper parameter tuning using a Bayesian optimizer with Keras tuner.^104^ The network information used in each case is shown in Table 2. For DDSBB, we apply the sampling limits of 1000, 2500, and 5000 samples for 2, 5, and 10 dimensions, respectively, to ensure a fair comparison with surrogate methods. The absolute and relative tolerance values were set to 0.05 and 0.01. The optimization is repeated 30 times with different sampling initializations to assess solution variability.

**Table 2:** Neural Network Configurations with Tanh and ReLU Activations

dimensionality	network architecture	activation	training data size	training epochs
2	2–100–100–1	Tanh	1000	4000
2	2–100–100–1	ReLU	1000	4000
5	5–40–30–20–1	Tanh	2500	5000
5	5–30–20–30–1	ReLU	2500	5000
10	10–20–20–20–20–1	Tanh	5000	6000
10	10–20–30–25–30–1	ReLU	5000	6000

**Figure 10:**
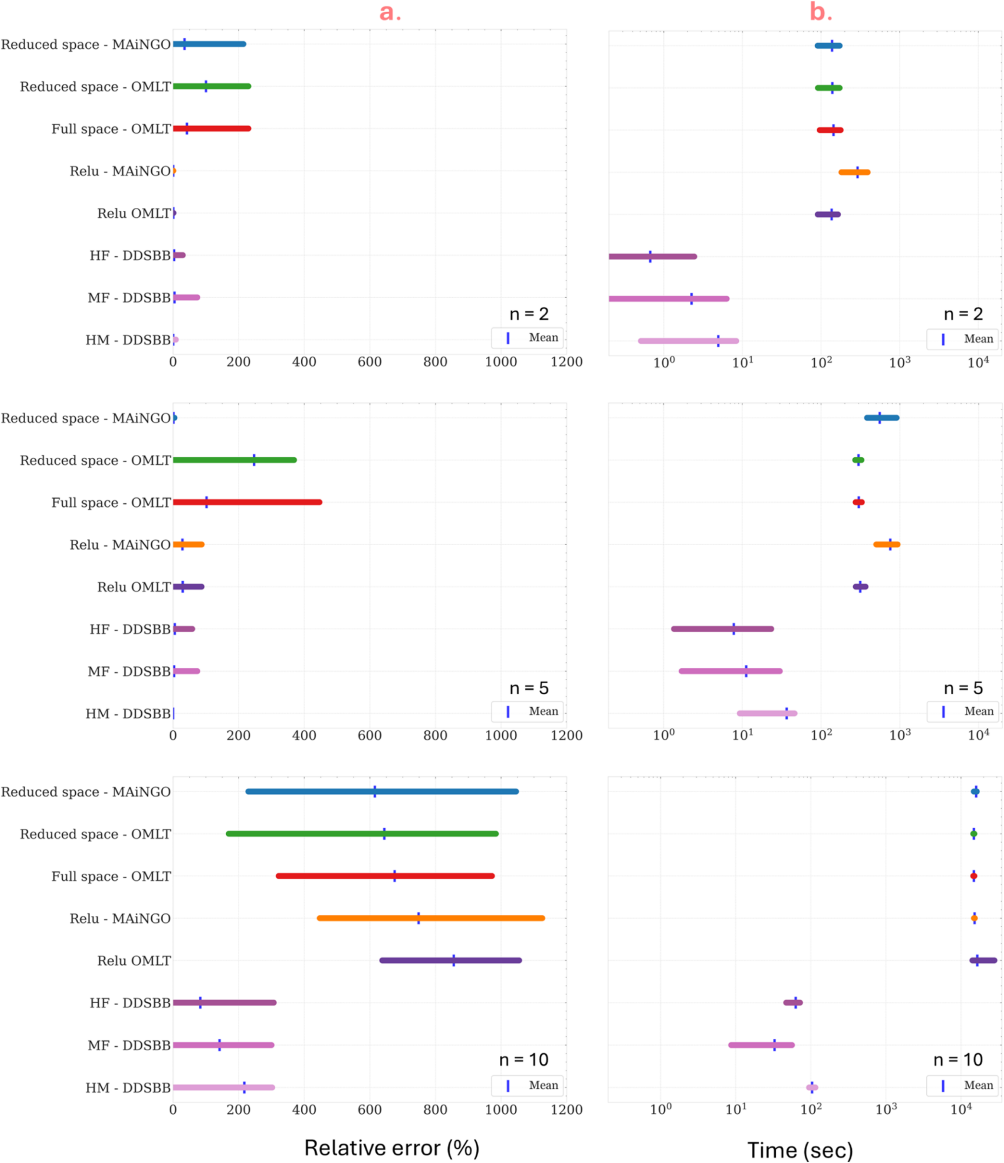
(a) Relative error of the discovered solution with respect to the true optimum. (b) The time requirements for modeling and optimization, for *a priori* samples surrogate-based optimization and adaptive-sampling-based optimization for 2, 5, and 10 dimensional Rastrigin function.

Figure 10 illustrates the relative error and CPU requirement plots for both *a priori* sampled surrogate optimization and adaptive sampling-based optimization with increasing dimensionality. In Figure 10a, the *x*-axis shows the relative error of the solution compared to the true optimum, while in Figure 10b, the *x*-axis represents the CPU requirements for optimization. The relative error increases with dimensionality for both methods, but the adaptive sampling approach consistently shows lower relative errors. As dimensionality increases, variations in sampling significantly impact the surrogate profile, resulting in greater variability in the solution for *a priori* sampled surrogate optimization. This further underscores the need for more data to train the surrogate models in higher dimensions. Additionally, we do not show the results with HM due to the resulting MINLP structure even with ReLU activations, because of the nonlinearity constraint from low-fidelity model. This requires an MINLP solver and very high CPU times for convergence as observed from the analysis in Section 3.1.

Among the three methods compared under the adaptive sampling approach, DDSBB with hybrid modeling outperforms the multifidelity and high-fidelity approaches for 2 and 5 dimensions. However, even though the relative error range is similar under all three DDSBB methods for the 10-dimensional function, the mean relative error for the HM method is higher than for the HF and MF methods. This discrepancy can be attributed to the architecture of the NN used for HM under DDSBB, which was set without hyperparameter tuning. In adaptive sampling, this NN architecture can overfit or underfit the data, particularly as the search region narrows with complex structures, affecting the optimization search. In contrast, the HF approach, which does not rely on any ML model for additional samples, shows comparatively lower variation. The MF approach uses SVR as the surrogate for additional low-fidelity samples. Since SVR structure relies on the data itself, it is less likely to overfit, resulting in a lower mean error.

While the higher mean error for the HM method in higher dimensions might seem problematic, this may be addressed in two ways. A simpler hyperparameter tuning with moderately complex NNs can address this issue or using a surrogate model that has an architecture dependent on the data and the quantity of the data itself like SVR or a Gaussian process model. For simpler cases, interpolating models like cubic splines can also be utilized. Additionally, dynamically changing the surrogate structure with adaptive sampling can also help. Overall, even at a dimensionality as high as 10, the adaptive sampling methods perform better than the *a priori* sampled surrogate optimization approach.

Figure 10b illustrates the time requirements for model training and optimization. For all methods under the *a priori* sampled surrogate optimization, the time displayed includes time requirements hyper-parameter tuning. For this analysis as well, the NN architecture with lowest training and validation errors was determined through hyperparameter tuning using a Bayesian optimizer with Keras Tuner.^104^ Both approaches show an increase in time requirements with dimensionality due to the complex function profiles in higher dimensions. This complexity necessitates longer training times for the surrogate methods for more intricate NN models. These NN models, when optimized, translate to more equations, constraints, and variables, thus requiring longer convergence times. The same trend is observed for methods with adaptive sampling optimization under DDSBB. Among the high-fidelity, multifidelity, and HM methods, the HM method takes the most time, followed by MF and HF methods. This higher time requirement is attributed to the more complex MFSM structure training in the HM-DDSBB method.

Furthermore, all methods under the adaptive sampling optimization consistently require at least an order of magnitude less time compared to the *a priori* sampled surrogate optimization. This shows efficiency and computational advantage of the adaptive sampling approach over the *a priori* sampled surrogate-based method.

### 3.3 Case Study on Temperature Vacuum Swing Adsorption of CO_2_

In the previous section, our analysis with the test functions demonstrated that hybrid modeling methods have the potential to improve the accuracy of surrogate models and robustness. In this section, we shift our focus to a chemical engineering case study on the temperature vacuum swing adsorption of CO_2_. The process simulator is simulated using gPROMS Model Builder V7.1.1. This model is a detailed partial differential-algebraic equations (pDAE) based model built to simulate the operation of a direct air capture (DAC) unit. There are 5 stages in the operation.

1. Adsorption: The air is blown into the system, and CO_2_ is absorbed onto the fibers.
2. Evacuation: Vacuum is applied to reduce non-CO_2_ component concentration.
3. Pressurization: Inject the steam to pressurize the system back to 1 atm.
4. Desorption: Keep injecting steam and open the outlet. The steam heats the fibers up, and the CO_2_ will be released from the fibers. At the outlet, the CO_2_-steam mixture can be collected.
5. Cooling: Vacuum is applied again. The water on the fiber in the system flash evaporates by pressure change, which takes the temperature of fibers down before the conducting with air to avoid severe degradation.

**Figure 11:**
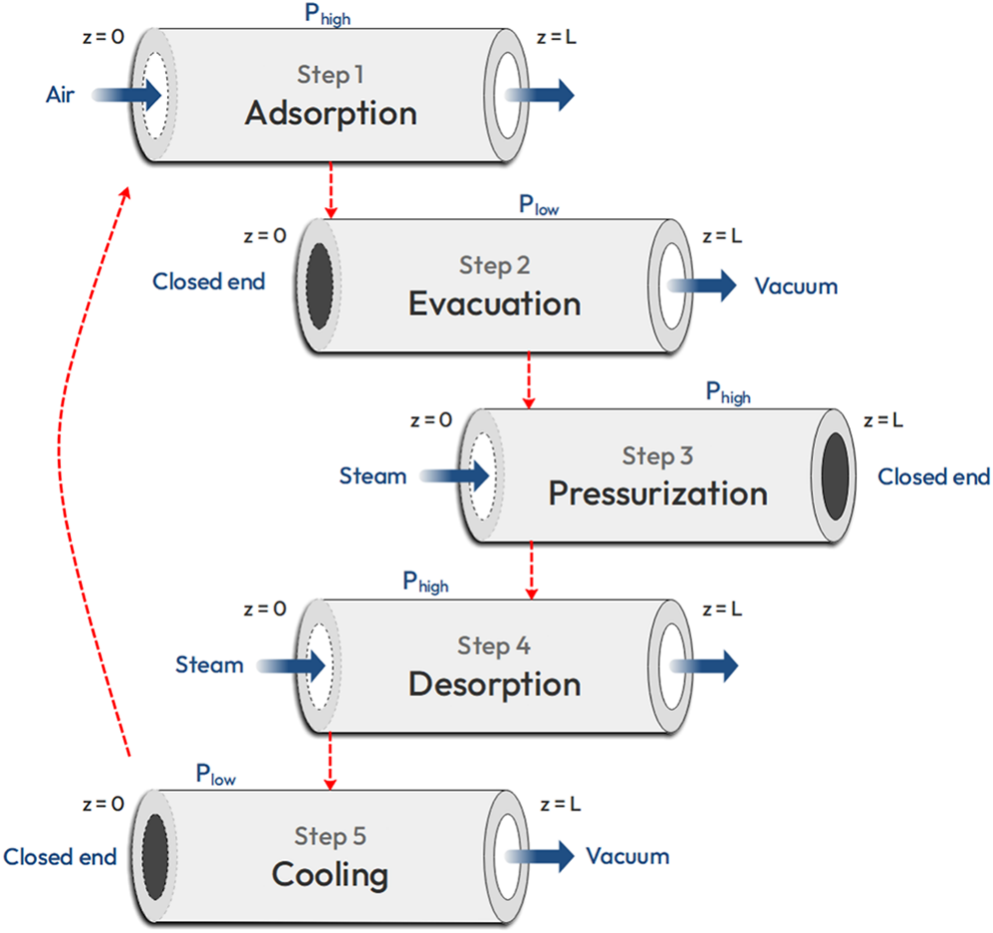
Overview of the steps involved in the TVSA model for DAC.

Figure 11 shows an overview of the steps involved in the TVSA system for the DAC process. The optimization goal is to minimize the operational cost per net CO_2_ captured (*OCNC*) over *N* future cycles. Decision variables include adsorption durations (*A*_*i*_) and desorption durations (*D*_*i*_) of all cycles. In this case study, *N* = 3. The relevant parameters, input and output variables for the simulation are outlined in Table 3. Detailed information on the optimization problem for the TVSA system with all the equations is included in Supporting Information – Section 6.

**Table 3:** Description of Input and Output Variables for TVSA System

	variable	description
input variables	*A*_1_	adsorption time of cycle 2
	*D*_1_	desorption time of cycle 2
	*A*_2_	adsorption time of cycle 3
	*D*_2_	desorption time of cycle 3
	*A*_3_	adsorption time of cycle 4
	*D*_3_	desorption time of cycle 4
output variables	OCNC	operational cost per net CO_2_ captured

#### 3.3.1 *A Priori* Sampling Surrogate-Based Optimization

The HF simulation is developed in gPROMS^109^ and it is integrated with Python using the gORUN API. For constructing the surrogate model, input-output pairs are generated to form training, validation, and test data sets using the HF simulation, employing a LHS design. The adsorption and desorption times range from 2000 to 8000 s and 1500 to 3000 s, respectively. The simulation is continuous with respect to the search space but failed to converge at some sample locations due to simulation or process infeasibility. To address this, the simulation was iteratively executed with a maximum of 2000 samples. In cases where the simulation failed to converge, a random value exceeding 300 was assigned to OCNC. Overall, the system is treated as black-box simulation with continuous inputs and outputs. At the end of sample generation, the collected data is divided into 1250, 500, and 250 data points for training, validation, and testing, respectively.

We then build a NN model using the training set. A two-layer architecture is employed, utilizing both “tanh” and “ReLU” activations. The number of nodes in the two layers is constrained between 30 and 60 to find the best configuration. This configuration is determined through hyperparameter tuning using a Bayesian optimizer with Keras Tuner.^104^ The maximum number of training epochs was set to 10000 and with a patience value set to 500 over the validation loss to avoid any overfitting. Based on the activation function, an optimization formulation in a nonlinear programming (NLP) or mixed-integer programming (MIP) problem with the corresponding surrogate model was solved with equation based solvers.

A low-fidelity simulation was developed to complement the HF simulation with reduced complexity and significantly faster computation times. For the HM analysis, the LF model was integrated as a black-box component within the error correction model. While HM can accommodate black-box components, optimization using equation-based solvers is not possible due to the absence of explicit input-output relationships within the LF model. To address this, an SVR model was constructed to approximate the LF simulation, thereby enabling equation-based optimization.

Input-output data for training and testing the SVR model were generated within the same search space as the HF simulation. A data set of 5000 LF data points was partitioned into training (3000 points) and testing (2000 points) sets. The SVR model was trained with tuned hyperparameters, resulting in *R*^2^ scores of 0.93 and 0.83 for the training and test sets, respectively. A detailed analysis in which we test other surrogate model candidates as the LF model is provided in Supporting Information – Section 7. The SVR-based LF model was subsequently integrated into the HM framework using a MFSM structure. The HM was trained with a maximum of 10,000 epochs, incorporating early stopping with a patience value of 500 over validation set to mitigate overfitting. Depending on the activation function, the resulting optimization problem was formulated as either a nonlinear programming (NLP) or mixed-integer nonlinear programming (MINLP) problem, which was solved using equation-based solvers to determine the optimal solution.

#### 3.3.2 Adaptive Sampling-Based Optimization

To implement adaptive sampling-based optimization using DDSBB, the gORUN API was employed to integrate the HF simulation as a black-box model within the DDSBB framework. The HF simulation calculates the output value of OCNC and is used by DDSBB during optimization. Absolute and relative tolerance values for the upper and lower bound gaps were set to 0.05, with a sampling limit of 2000. For comparison with HM, the LF simulation was directly coupled with the MFSM pseudocode outlined in Section 2, by skipping the first two steps of Algorithm 1.

**Figure 12:**
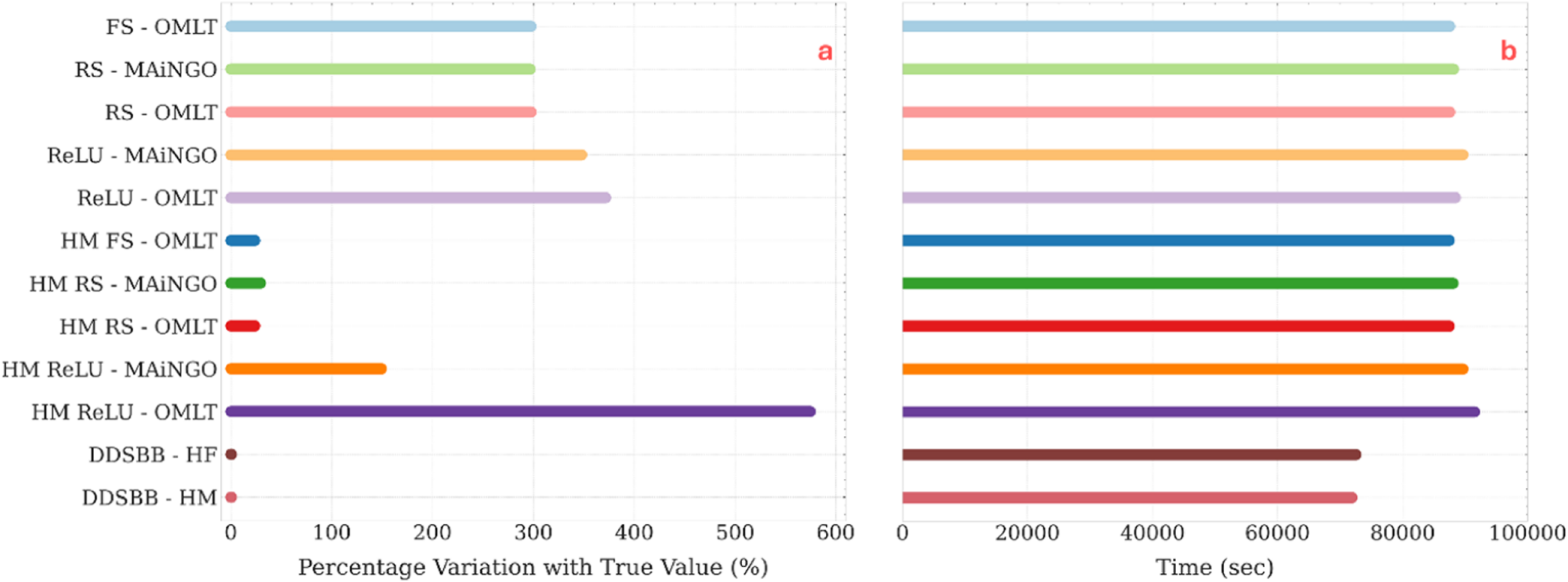
TVSA case study: (a) The percentage variation between reported (from optimization) and the true objective values (corresponding true simulation outputs), (b) time requirements for modeling and optimization for both surrogate methods and adaptive sampling methods.

Figure 12a presents the relative error between predicted optima and the true output determined by HF simulation using the predicted solutions. A discrepancy between predicted optima and true output is evident for all surrogate modeling methods with a priori sampling. Although both black-box and HM methods exhibit this deviation, the latter demonstrates smaller deviations. The solutions found as optimal across all methods are generally comparable, except for the solution identified using the full-space formulation with HM. The NN architecture employed tanh activations for both the reduced-space and full-space formulations, while ReLU activations were used for ReLU-MIP formulations.

Although the NN model’s optimal solution was identified, it may deviate from the true output value or lead to a failed simulation (executed with the identified optimum), as observed in Figure 12a. Given the limited feasible space of the simulation, several challenges arise in locating the optimal solution. First, training the NN model to achieve high accuracy is inherently difficult and requires rigorous hyperparameter tuning. Despite these challenges, NN models with *R*^2^ scores exceeding 0.99 were successfully trained and used in this case study.

The results in Table 4 indicate that there are several local optima identified by the different strategies. Several factors may contribute to the observed behavior. First, it indicates that due to the cyclic operation and competing effects of operating conditions, the true objective function for this simulation is nonconvex, with multiple local minima. Moreover, local optima may arise due to the presence of noisy outputs and derivatives resulting from the surrogate fit itself. Although multistart IPOPT was again used to improve the chance of converging to global solutions, we observe that each approach leads to different local solutions. What is surprising is that the solutions obtained by all nonadaptive methods lead to varying adsorption and desorption times across cycles, and there is no consistent trend when comparing the solutions. A possible physical explanation for differences between cycles is that the first cycle starts with fresh sorbent (*q* = 0 mol-CO2/kg-sorbent), but the sorbent can never reach a low loading during the desorption, which means future cycles cannot have the same starting conditions. However, when looking at the consistency in the solution obtained by the adaptive optimization approach, another plausible explanation for such differences is the convergence to local optima. This is more pronounced in the full-space formulation with HM, highlighting the need for a global solver to locate global solutions effectively. For the ReLU formulations, a similar trend is observed. It is important to note that HM with ReLU formulations results in a MINLP problem unless the LF model used for HM is a linear model and might result in increased time requirements for optimization.

**Table 4:** Input, Objective Values, and Feasibility Information of the Optimum for TVSA System

method	Optimum x (*A*_1_, *D*_1_, *A*_2_, *D*_2_, *A*_3_, *D*_3_)	Predicted Optimum y (*OCNC*_*predicted*_)	True Optimum y (*OCNC*_*true*_)	solution feasibility
Full space - OMLT	2002, 1501, 2002, 3000, 2002, 1501	93.429	292.182	feasible
Reduced space - MAiNGO	2005, 1501, 2816, 2707, 7997, 2994	92.494	279.75	infeasible
Reduced space - OMLT	2002, 3000, 2002, 3000, 7998, 3000	92.214	283.011	infeasible
ReLU - MAiNGO	2002, 1501, 7981, 1501, 3309, 2965	93.261	259.51	infeasible
ReLU - OMLT	2002, 2280, 7690, 1730, 7998, 1865	87.756	280.48	infeasible
HM Full space - OMLT	4874, 1501, 3826, 2941, 3440, 1533	243.747	104.42	feasible
HM Reduced space - MAiNGO	2006, 1501, 5008, 1726, 6743, 2975	94.252	283.89	feasible
HM Reduced space - OMLT	2002, 2251, 5653, 3000, 6646, 2798	96.008	138.51	feasible
HM Relu - MAiNGO	2002, 1501, 2646, 1501, 2161, 3000	85.57	157.251	feasible
HM Relu - OMLT	2002, 1501, 2362, 1501, 2002, 3000	84.76	169.767	feasible
DDSBB - HF	5750, 1500, 5000, 1500, 5000, 1500	89.48	89.48	feasible
DDSBB - HM	5750, 1500, 5000, 1500, 5000, 1500	89.48	89.48	feasible

Second, even with the model with the lowest error after parameter tuning, predicting all values accurately across the entire search space remains challenging. This difficulty stems from the limited data available due to the expensive high-fidelity simulations and the limited feasible space of the formulation. A discrepancy can arise when the search space in the high-fidelity simulation contains infeasible regions, but the NN approximation assumes a continuous feasible space. As a result, when this approximation is employed in the optimization formulation, it may yield a solution that appears to be a feasible optimum but is, in reality, infeasible with respect to the true simulation. Third, constructing surrogate models that are accurate near the boundaries of the search space is challenging and heavily depends on the quality of the sampling. Consequently, when optimization drives the search toward boundaries, an inaccurate surrogate model can result in suboptimal solutions. This highlights the advantage of an adaptive sampling strategy, as it allows for better exploration of these critical regions.

All the solutions except those from the full-space formulation using black-box a priori sampled surrogate methods resulted in failed simulations. This is not the case for methods using HM, where all identified solutions led to feasible simulations. It is important to note that the network architecture for both black-box and HM NN models was kept consistent, but the HM model also incorporated an additional input from the SVR LF model. This demonstrates that an LF model can enhance solution quality and that having an accurate LF model for equation-based solvers is beneficial for optimization. However, an inaccurate LF model can degrade the solution quality. Therefore, the complexity and accuracy of the LF model should be carefully considered.

Because the adaptive sampling methods rely directly on the sampled data, there is no discrepancy between the reported and true solutions, as shown in Figure 12a. In fact, the solution found using this approach has the lowest objective value. Both the black-box and HM methods with adaptive sampling yielded the same optimal solution. Furthermore, since locating the solution does not require equations from the LF or HF models, the existing LF simulation can be directly used for HM with the adaptive sampling approach.

In Figure 12b, we present the time requirements for optimization for both approaches. These time requirements encompass sampling, modeling, and optimization times. While the majority of the time is attributed to the sampling of the expensive HF simulation, it is significantly lower for the adaptive sampling approaches by at least 15,000 s. Within the adaptive sampling approach, the time requirement for the HM method is relatively lower than that for the black-box method. Among the methods compared under surrogate optimization with *a priori* sampling, the ReLU formulation with HM required the most time, as the corresponding optimization formulation is a MINLP. The input variables and objective values obtained through optimization for both approaches are detailed in Table 4.

### 3.4 Case Study on Extractive Distillation of *n*-Heptane and Toluene

**Figure 13:**
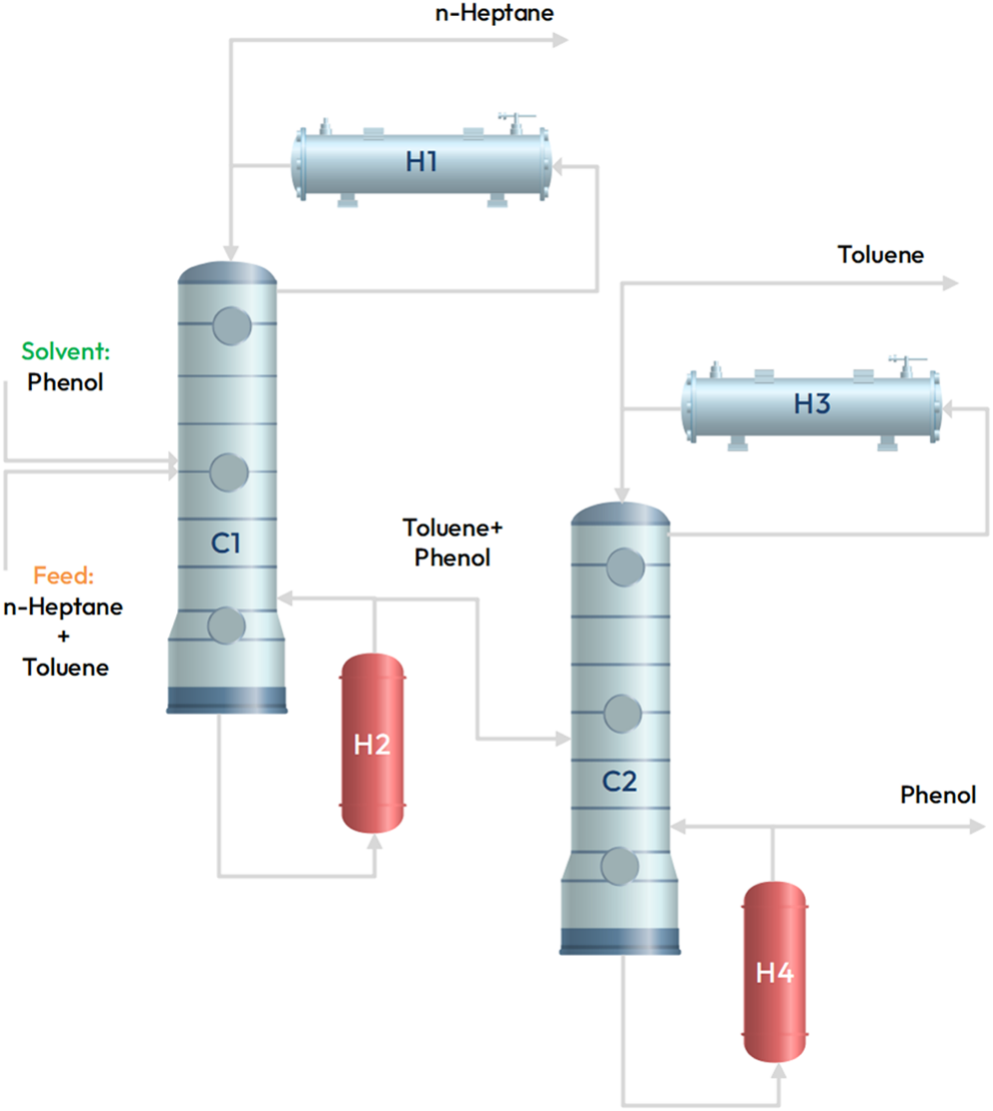
Process flow diagram for extracting toluene from *n*-heptane using phenol solvent.

The last case study we consider is on the extractive distillation of *n*-Heptane and Toluene. The goal of the process is to minimize the energy costs of an extractive distillation process that separates toluene from *n*-heptane. The process simulator is simulated using Aspen Plus V.11, and the case study is taken from the literature.^110^ The process employs phenol as an extractive solvent to separate the azeotropic mixture of toluene and *n*-heptane. Figure 13 illustrates the process flow diagram, featuring two distillation columns *C*_1_ and *C*_2_. The feed to the column is a fixed equal mole ratio mixture of toluene and *n*-heptane, while the solvent stream comprises the solvent phenol. Column *C*_1_ separates *n*-heptane, and its bottoms are fed to column *C*_2_, which separates toluene. The purified final products are obtained in the distillate streams of columns *C*_1_ and *C*_2_. The bottom stream of the column contains recycled solvent phenol.

The optimization goal is to minimize the heat duty in the condensers and reboilers of columns *C*_1_ and *C*_2_. Decision variables under consideration include the amount of extractive solvent in the solvent stream, as well as the reflux ratios and distillate rates in columns *C*_1_ and *C*_2_. Additionally, the two product streams, i.e., distillate streams of columns *C*_1_ and *C*_2_, must meet purity specifications. The relevant parameters, input and output variables for the simulation are outlined in Tables 5 and 6.

**Table 5:** Description of Parameters for the Optimization Formulation of Extractive Distillation Case Study

parameter	description
α_*c*_	cost coefficient for condenser
α_*r*_	cost coefficient for reboiler
*p*_*n*_	purity requirement of *n*-heptane
*p*_*t*_	purity requirement of toluene

**Table 6:** Description of Input and Output Variables for Extractive Distillation Process Simulation

	variable	description
input variables	*F*_*s*_	mole flow of phenol in the solvent stream
	*D*_1_	distillate rate of column *C*_1_
	*r*_1_	reflux ratio of column *C*_1_
	*D*_2_	distillate rate of column *C*_2_
	*r*_2_	reflux ratio of column *C*_2_
output variables	*x*_*n*_	mole fraction of *n*-heptane in the *C*_1_ distillate stream
	*x*_*t*_	mole fraction of *n*-heptane in the *C*_2_ distillate stream
	*E*_1_	condenser duty of column *C*_1_
	*E*_2_	reboiler duty of column *C*_1_
	*E*_3_	condenser duty of column *C*_2_
	*E*_4_	reboiler duty of column *C*_2_

For the analysis we set the cost coefficients of condenser and reboiler duties to α_*c*_, α_*r*_ = 3*e*^–4^, 2*e*^–4^ respectively, and the purity requirements for both *n*-heptane and toluene as *p*_*i*_ = 0.9 ∀ *i* ∈ [*t*,*n*]. The objective is to minimize the energy cost and is formulated as follows:
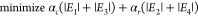
17
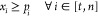
18
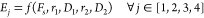
19
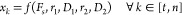
20

#### 3.4.1 *A Priori* Sampling-Based Surrogate Optimization

In order to construct the surrogate model, input-output pairs were generated to form training and validation data sets using the Aspen simulation, employing a LHS design. The simulation is treated as continuous function with respect to the variables and the upper and lower bounds for the input variables are detailed in Table 7. The simulation, constrained by a sampling limit of 3700, was run iteratively. However, it failed to converge at some points in the search space due to infeasibilities in mass balances. Ultimately, a total of 3550 input-output pairs were generated and were divided into 3000, 250, 300 data points for training, validation and testing respectively. In order to incorporate the constraints while formulating the surrogate model, we make use of the combined objective function formulation by merging the equations, eqs 17 and 18 as shown in eq 21.
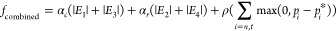
21We set the value of ρ to 10^4^ to balance the weights of the two terms in equation and *p*_i_^*^ represents the purity of the product *i*, ∀ *i* = *n*, *t* for a given input. Utilizing eq 21 for training the surrogate model helps in accommodating the constraint violation. The term ρ(∑_*i*=*n*,*t*_ max(0, *p*_*i*_ – *p*_i_^*^)) contributes a value of zero to the objective function when the constraints are satisfied, and it adds a scaled value of a violation to the objective function when the constraints are not met.

**Table 7:** Lower and Upper Bounds for the Input Domain

variable	lower bound	upper bound
*F*_*s*_	20.0 kmol/h	100.0 kmol/h
*r*_1_	1.0	20.0
*D*_1_	20.0 kmol/h	80.0 kmol/h
*r*_2_	1.0	20.0
*D*_2_	20.0 kmol/h	80.0 kmol/h

To utilize the MFSM structure with the hybrid modeling approach, a LF model is required. Since we do not have a pre-existing LF model for this case study, we adopt a two-step model correction workflow proposed in,^42^ as discussed in Algorithm 1 in Section 2. In the first step, a portion of the data set is used to train a low-fidelity SVR model, approximating the combined output from eq 21. Subsequently, a NN model is employed in the second step to correct the errors introduced by the SVR model, to match the predictions with the HF output.

The selection of SVR as the LF model is justified by its lower complexity compared to other surrogate models. A detailed analysis which we test other surrogate model candidates as the LF model and their performance is provided in Supporting Information – Section 7. A NN model is then developed to build the composite structure of MFSM using the training data set. A three-layer architecture is employed, incorporating both “tanh” and “ReLU” activation functions. The number of nodes in the hidden layers is optimized within a range of 10 to 30 to identify the best configuration. This optimal configuration is determined through hyperparameter tuning using a Bayesian optimizer with Keras Tuner.^104^ The training process is set to a maximum of 10,000 epochs, with an early stopping criterion based on validation loss, utilizing a patience value of 500 to prevent overfitting. Depending on the activation function, the optimization problem is formulated as either a NLP or MIP problem and solved with equation-based solvers to determine the optimum. For the HM approach, we use a NN with the same architecture, incorporating the output of the LF SVR model as an additional input. This MFSM is then optimized by incorporating the SVR model equation as an additional constraint in the optimization formulation.

#### 3.4.2 Adaptive Sampling-Based Optimization

For adaptive sampling-based optimization, we integrate the Aspen-based HF simulation with DDSBB using the Aspen-Python API to facilitate the adaptive sampling process. The HF simulation serves as the underlying black-box model, providing the output *f*_combined_ from eq 21 for the simulation. The absolute and relative tolerance values for the upper and lower bound gaps are set to 0.05, and the sampling limit is set to 3550. For the hybrid modeling approach, we implement the same Algorithm 1 outlined in section 2. The HF simulation is then optimized by employing the MFSM approach to locate the optimum solution.

**Figure 14:**
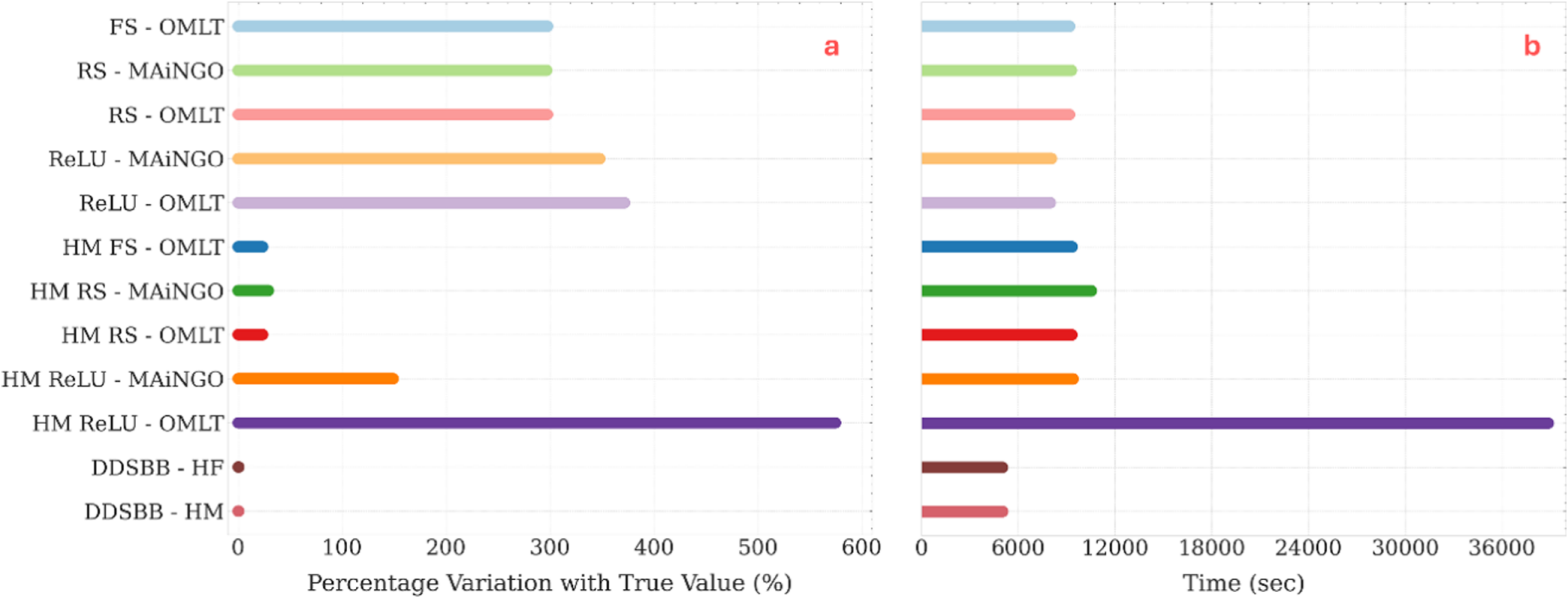
Extractive distillation case study: (a) The percentage variation between reported (from optimization) and the true objective values (corresponding true simulation outputs), (b) time requirements for modeling and optimization for both surrogate methods and adaptive sampling methods.

The optimization results for both approaches are presented in Table 8. Table 8 shows the purity values for *n*-heptane and toluene calculated with the optimum solution obtained using both the black-box and MFSM model correction methods. The optimization results for both approaches are presented in Figure 14. Similar to the previous case study, a relative error between the predicted optimum and the true simulation output at the incumbent optimum is calculated. A clear discrepancy exists between predicted and true optima for all surrogate modeling methods that employ *a priori* sampling. Although both black-box and hybrid modeling methods suffer from this deviation, hybrid modeling methods exhibit a smaller discrepancy.

**Table 8:** Input, Objective Values, and Feasibility Information of the Optimum for Extractive Distillation System

		optimum *y*		composition		
method	optimum *x* (*F*_*s*_, *r*_1_, *D*_1_, *r*_2_, *D*_2_)	(*f*_combined_^predicted^)	(*f*_combined_^true^)	(*x*_*n*_)	(*x*_*t*_)	solution feasibility
Full space - OMLT	100.0, 3.7, 52.7, 1.0, 45.4	69.74	276.97	0.921	0.969	feasible
Reduced space - MAiNGO	99.9, 3.7, 52.7, 1.0, 45.4	69.88	276.98	0.921	0.969	feasible
Reduced space - OMLT	100.0, 3.7, 52.7, 1.0, 45.4	69.74	276.97	0.921	0.969	feasible
ReLU - MAiNGO	21.0, 5.8, 50.0, 1.0, 53.9	86.65	387.63	0.981	0.917	feasible
ReLU - OMLT	20.1, 5.8, 50.1, 1.0, 53.8	82.91	390.56	0.980	0.917	feasible
HM Full space - OMLT	92.3, 4.6, 51.6, 1.0, 36.5	247.88	305.21	0.963	0.992	feasible
HM Reduced space - MAiNGO	90.0, 5.1, 50.0, 1.0, 41.7	258.41	332.31	0.990	0.989	feasible
HM Reduced space - OMLT	92.3, 4.6, 51.6, 1.0, 36.5	247.88	305.21	0.963	0.992	feasible
HM Relu - MAiNGO	100.0, 5.3, 50.0, 1.0, 33.3	133.82	332.93	0.990	0.988	feasible
HM Relu - OMLT	90.1, 2.4, 62.6, 3.3, 30.8	64.37	434.01	0.781	0.964	infeasible
DDSBB - HF	86.1, 2.7, 55.1, 3.1, 28.0	341.51	341.51	0.830	0.851	infeasible
DDSBB - HM	41.6, 3.6, 50.5, 1.4, 36.7	264.09	264.09	0.919	0.904	feasible

The purity constraints for *n*-heptane and toluene are satisfied by all the methods compared, except for the ReLU formulation with HM and the black-box adaptive sampling approach. It is important to note that when the nonlinear SVR LF model is used with HM, the resulting MINLP problem becomes more complex. To address this, the global solver BARON was employed, which yielded a better predicted optimum. However, when this predicted optimum was tested in the HF simulation, the true output resulted in an infeasible solution with respect to the purity constraints.

As discussed in the previous case study, these discrepancies can arise when NN models assuming convex feasible spaces, are used to approximate function profiles with intermittent infeasibilities. This can lead to surrogate model solutions, but in reality, could be infeasible solutions in true simulation space. Unfortunately, such scenarios may be unavoidable and overcoming this challenge requires more data to be able to represent the system or the simulation as closely as possible. A similar issue can arise with sampling-based techniques. Infeasibilities in the search space can also impede adaptive sampling methods heuristics, potentially leading to suboptimal solutions, as observed with the black-box DDSBB solution. That said, these heuristic behaviors can be managed by implementing various types of penalties when the simulation fails, which can help guide the search process more effectively.^110^

In Figure 14b, the time requirements for optimization for both approaches are shown. It is also evident that the time requirements for modeling and optimization for the surrogate methods with *a priori* sampling is almost twice as the adaptive sampling methods. This is primarily attributed to the sampling costs and the time required to train the surrogate model with the 3550 samples collected. On the other hand, both adaptive sampling methods converge under 1500 samples, with a comparable objective value and consequently, lower time requirements. The input variables, objective values and the feasibility information obtained through optimization for both methods are detailed in Table 8.

## 4 Discussion

There are several aspects and findings of this study that are worth further discussing. One of the approaches using ML techniques for simulation optimization involves conducting extensive *a priori* sampling, followed by fitting the most optimal surrogate model. Subsequently, this surrogate model is integrated into an optimization formulation to locate the optimal solution. This strategy proves particularly valuable (or sometimes the only feasible approach) in cases where further data collection from the simulation is impractical, or when the surrogate is embedded within a larger optimization problem. But there are a few challenges as we discussed in the paper in globally optimizing the surrogate model.

First, building an accurate surrogate model that is robust to the variations in the data and with limited availability of data is a challenge.^111^ This can be attributed to the fact that the ML surrogates have several data-dependent parameters. So, the changes in the data affect the model parameters and hence the incumbent optimal solutions, which is shown through the results in this paper. Several factors influence the effectiveness of a surrogate model, including its complexity and training duration. Selecting an appropriate surrogate model requires careful parameter tuning; a model that is too simple may underfit the data, while an overly complex model can overfit the data. Ideally, a surrogate model should strike a balance between accuracy and complexity. While this may sound easy, in reality, hyper-parameter tuning may take much longer to find the best possible structure to fit the data by checking all the possible combinations. In general, when looking to improve the accuracy of a surrogate model such as a NN, one may explore two primary options: increasing the depth (number of layers) or increasing the width (number of neurons per layer). While deeper networks can capture more complex nonlinearities, they often face challenges such as vanishing or exploding gradients, increased computational cost, and a higher risk of overfitting.^112^ Similarly, while wider networks may be easier to train, they are also susceptible to overfitting and can lead to very large and complex optimization formulations (comparative analysis shown in Supporting Information – Section 1).

Additionally, the quantity of training samples significantly affects the quality of the solution. Insufficient samples may not adequately represent regions containing global solutions, leading to potential underfitting or overfitting, and thus, suboptimal solutions upon optimization. Although increasing the number of training samples generally helps mitigate this issue, it may not always be feasible due to the computational expense involved in collecting these samples, particularly in nonlinear systems. As observed in Figure 9 shown in Section 3, and Tables S2–S7 from Supporting Information, this error and the corresponding variability in the error decreases with increase in the amount of sampling. For example, the average error across 30 repetitions, between the reported solution and true optimum changed as follows:

1. **Reduced Space MAiNGO:** Increasing the sample size from 100 to 1000 resulted in an 80% reduction in average error, improving from 7.80 to 1.56%.
2. **ReLU-MAiNGO:** Increasing the sample size from 100 to 1000 led to a 73% reduction in average error, decreasing from 7.25 to 1.95%.
3. **ReLU-OMLT:** Increasing the sample size from 100 to 1000 resulted in a 72.5% reduction in average error, improving from 7.16 to 1.97%.

Furthermore, in cases involving nonlinear systems, adding more samples generally improves the accuracy of capturing the function profile as observed in the results. However, it is important to recognize that in regions with smoother or linear behavior, increasing the sample count may not significantly improve the surrogate model’s fit. This underscores the importance of employing adaptive sampling to optimize data collection and model accuracy effectively.

Second, formulating the surrogate model for optimization itself is a challenging task. While state-of-the-art tools like OMLT and MAINGO have been developed to seamlessly facilitate these formulations, the choice of surrogate is still limited to a few options. As software packages or toolkits for implementing optimization formulations become more standardized, we anticipate the selection of the surrogate models and parameter tuning to be increasingly more streamlined. We also observed that the type of formulation used and the choice of the optimization solver matter when it comes to locating the global optimum. Several factors contribute to this such as the complexity, nonlinearity, and nonconvexity of the surrogate model. From the variety of options such as reduced-space, full-space and ReLU formulations etc., choosing the type of formulation would depend on the end use of the surrogate model (e.g., embedded within a larger formulation or not), solver availability and available computational resources.

Ideally, in order to locate the global solution, one would want to incorporate as much information as possible into the formulation and use a global solver for optimization. This might seem like it can be easily achieved, but two main challenges should be kept in mind. Increase in the dimensionality or the surrogate complexity increases the optimization problem complexity drastically, making the computational and time requirements needed for global optimization the most time-consuming step. Our analysis under the Multi-Gauss case study in Figure 9 illustrates that at lower sample sizes, ReLU-based methods are notably more time-consuming compared to reduced space formulations during optimization. However, this time gap narrows as the sample size increases. For instance, with 100 samples, ReLU methods—are significantly slower, taking over 8x times longer on average than Reduced Space methods. At 1000 samples, the gap decreases, but ReLU methods still require approximately 1.5–2x times more.

Third, there also exist cases where even with a tuned surrogate model that has the least error, achieving accurate predictions across the entire search space remains elusive. This limitation can be attributed to the scarcity of high-fidelity simulation data and/or the presence of nonconvexity in the feasible space. This can lead to surrogate-based optimal solutions that are in reality infeasible. Moreover, constructing surrogate models that accurately represent the behavior near the boundaries of the feasible space is an important consideration, especially since often optimal solutions are near or on boundaries. The quality of the sampling process significantly influences the surrogate model’s ability to capture the boundary and the nonlinearity of the high-fidelity simulation. When optimization drives the search toward these critical regions, an inaccurate surrogate model can lead to suboptimal solutions. Our analysis with the two engineering cases on extractive distillation and direct air capture systems shows the challenges associated with such cases. This underscores the importance of adaptive sampling strategies, which can effectively explore these critical regions.

An alternative strategy involves using the system or simulation iteratively for optimization. In such scenarios, an adaptive sampling optimizer is suitable. Although surrogate modeling can incorporate adaptive sampling in an iterative manner, leveraging acquisition functions to estimate potential improvements in the objective function requires continuous model fitting which can be costly. Conversely, employing an adaptive sampling strategy with a sampling-based optimizer, like the DDSBB algorithm discussed in our paper, offers several advantages. This method avoids the direct fitting of models to data, thus eliminating the associated costs. Our analysis using this approach demonstrated its robustness to data variations and its ability to achieve global solutions with reduced sampling requirements. Adaptive sampling methods provide superior exploration of the solution space relative to *a priori* sampling methods. For example, in the same Multi-Gauss case study, for HF-DDSBB, increasing the sample size from 100 to 1000 reduced the average error by 73%, improving from 1.33 to 0.36%. Similarly, MF-DDSBB showed a 91% reduction in average error, decreasing from 1.02 to 0.09%.

Given an identical number of samples, adaptive methods more effectively capture the profile of the function or simulation, particularly in regions near local and global optima. This improved representation enables the optimizer to more reliably identify global solutions, even in scenarios where data reinitializations occur. Additionally, since the underlying simulation is utilized solely for sampling and optimization, variations in data or sampling design do not affect the stability of the solution. Furthermore, we also noticed that since the simulation is adaptively sampled, sampling costs are reduced greatly and hence the time requirements. The average time requirements for adaptive sampling remained consistently low, at least an order of magnitude lower than those of surrogate methods.

Whenever there is a low-fidelity model (i.e., a surrogate model or semimechanistic model with lower accuracy that pre-exists), it can be used to construct hybrid models that are more accurate even with lower data availability. As seen from the case study results, these hybrid models are also more robust toward the changes in the data. These hybrid models can serve as sources of additional data and can aid in the optimization process for the adaptive sampling methods. Our analysis with the four case studies in the paper shows that HM combined with adaptive sampling methods further improved the solution quality (and had the least variability in the solution among all the approaches we compared), albeit increasing the optimization times slightly. For example, in the 100 samples case under Multi-Gauss case study, applying hybrid modeling significantly reduced average errors across methods, with reductions ranging from 13.5 to 89.4%. The most notable improvement was observed in ReLU-MAiNGO, where the error decreased by 89.4% (average error from 7.25 to 0.77%), while other methods saw reductions between ∼10% to ∼30%. For the adaptive methods, although the decrease in error was observed, it was not significant since the error was already low.

Furthermore, using hybrid modeling can help in improving model accuracy and robustness, and this was demonstrated in higher dimensions and multimodal systems. However, several potential challenges must be carefully considered before adopting the hybrid modeling route. Achieving higher accuracy may come at a cost of increased model complexity and resource demands, which might not always justify the effort, especially in cases where the improvement in accuracy is marginal. Therefore, it is crucial to carefully weigh the benefits of hybrid modeling against its potential drawbacks. We also observed from the results that in higher-dimensional cases (e.g., 10 dimensions), HM in adaptive sampling methods can become inefficient and might result in overfitting if not properly tuned as the search space is adaptively reduced. Tuning the hybrid model or using surrogate models that have a structure dependent on the data and the quantity of the data itself like SVR or a Gaussian process model can help in such cases. Future directions can include developing dynamic surrogate structures combined with low-fidelity models to create effective hybrid models for adaptive sampling methods.

Lastly, it is also important to note that in this study, both the LF and HF simulations operate within the same input space, and the model correction MFSM structure we use is effective for such scenarios. However, there exist instances where LF and HF models do not share the same input space, making the direct application of this MFSM structure impractical. Addressing these cases presents an opportunity for future research. Additionally, in this paper, we focused on a common engineering scenario—where a low-fidelity model complements a high-fidelity simulation—and employed the MFSM framework to construct composite models. While extending our analysis to include comparisons with other frameworks is beyond the scope of the current work, it represents another promising direction for future research.

## 5 Conclusions and Future Work

In this study, we evaluate two distinct data-driven methodologies commonly used for optimizing black-box functions and complex simulations. We focus on integrating hybrid modeling to improve the robustness and efficiency of the optimization process. Through mathematical case studies and engineering case studies on extractive distillation and temperature vacuum swing adsorption, we investigate the impact of hybrid modeling. The first methodology examined is surrogate-based optimization with *a priori* sampling, where data is collected beforehand, and a representative surrogate model is developed, formulated, and optimized using equation-based solvers in three different formulations: reduced space, full space, and ReLU. This approach revealed that hybrid modeling enhances the robustness of the surrogate model and reduces the variability of the optimum solution, even with fewer samples. Although hybrid modeling improves surrogate quality and robustness, it also increases the time required for optimization.

The second methodology explored is adaptive sampling-based optimization, where surrogate models serve as additional sources of samples within an adaptive sampling algorithm. We assessed three strategies within this framework: high-fidelity only, multifidelity, and hybrid modeling, and compared these with surrogate methods utilizing *a priori* sampling. Our findings indicate that adaptive sampling-based optimization is more efficient and robust in handling variations in sampling, sample quantity, and dimensionality than *a priori* sampled surrogate-based optimization methods. This efficiency is further enhanced by integrating multifidelity and hybrid modeling methods, which leverage low-fidelity models and surrogates for additional samples. In comparison, while adaptive sampling optimization reduces the need for extensive hyper parameter tuning, training and validation of universally accurate surrogate models, *a priori* surrogate-based optimization proves advantageous when a predictive surrogate model can be obtained and when frequent optimization or embedding within larger formulations are required.

## Supporting Information Available

Hybrid modeling with MINLP formulations, comparing different architectures of neural network surrogates, and a comparison of hybrid modeling integrated adaptive sampling method DDSBB with other widely used adaptive sampling methods (PDF)

## Abbreviations

HF: High-fidelity
LF: Low-fidelity
MF: Multifidelity
ML: Machine learning
HM: Hybrid modeling
MFSM: Multifidelity surrogate model
OMLT: Optimization and Machine Learning Toolkit
MAiNGO: McCormick-based Algorithm for mixed-integer Nonlinear Global Optimization
DDSBB: Data-driven spatial branch-and-bound
NN: Neural network
RS: Reduced space
FS: Full space
ReLU: Rectified Linear Unit
CFD: Computational fluid dynamics
SVR: Support vector regression
GPR: Gaussian process regression
UB: Upper bound
LB: Lower bound
MG: Multi-Gauss
LHS: Latin hypercube sampling
MSE: Mean squared error
MILP: Mixed integer linear program
NLP: Nonlinear program
MINLP: Mixed integer nonlinear program
MIP: Mixed-integer programming
IPOPT: Interior Point Optimizer
TVSA: Temperature vacuum swing adsorption
OCNC: Operational cost per net CO_2_ captured
DAC: Direct air capture
