# Supplementary material for: Effects of Surrogate Hybridization and Adaptive Sampling for Simulation-Based Optimization
Source: Ind Eng Chem Res. 2025 Apr 15;64(18):9228–51. doi: 10.1021/acs.iecr.4c03303 (PMC12063061; doi:10.1021/acs.iecr.4c03303)
Supplement: Supplementary file 1 — ie4c03303_si_001.pdf [file ie4c03303_si_001.pdf]

Supporting Information

Effects of Surrogate Hybridization and Adaptive Sampling for Simulation-Based Optimization

*Suryateja Ravutla [a], Andrew Bai [a], Matthew J. Realff [a], and Fani Boukouvala *[a]*

[a] Department of Chemical and Biomolecular Engineering, Georgia Institute of Technology, Atlanta, GA 30332 USA

* Corresponding Author Email: fani.boukouvala@chbe.gatech.edu

1. Effect of surrogate architecture (deep vs shallow) and training time on the variability of optimum solution

To investigate the impact of the surrogate architecture and complexity solution variability, we iterated through the process outlined in Figure 5 (main text) thirty times with two different sample sizes: 100, and 500 samples. In each run of the 30 repetitions, the sampling design undergoes a modification with random initialization based on LHS design, while maintaining a constant number of samples. The neural network's architecture remains unchanged, but it is retrained using the updated sampling design to minimize fitting errors and identify the best fit. We consider two neural network architectures: a wider neural network with two layers and a deeper neural network with four layers. configuration was determined through hyperparameter tuning using a Bayesian optimizer with Keras Tuner. Table S1 shows the architecture

information of neural networks used. Figure S1 and Figure S2 present the results depicting the influence of increasing sample sizes on solution variability with the two architectures considered. The variability is still observed with both the architectures, for both case of samples, although it decreases as the sample number increases from 100 to 500 samples. These results follow a similar trend as seen in the main text. The function profile is captured more effectively with a larger number of samples, and this reduces the impact of variation in the sampling design on the model parameters. Consequently, when the surrogate model is trained, the associated variability in the model parameters diminishes, leading to reduced variability in the solution.

Similarly, increasing the training time may lead to improvements in the surrogate model in some cases, as seen in Figures S1 and S3, where the number of neural network training epochs was increased from 4,000 to 7,500. Both the architectures, wider and the deeper network convergence to the global solutions improved. However, making a definitive claim about such improvements is challenging because the surrogate model's performance is highly dependent on the data. If the samples do not uniformly cover the function or the underlying simulation profile, extended training may not enhance the surrogate fit. Instead, it can lead to overfitting, as the model begins to memorize the training data rather than generalizing well. Consequently, when an overfitted surrogate model is used in an optimization formulation, the resulting optimum is unlikely to be robust to changes in problem data or input conditions. It is essential to verify or test the surrogate model on a different set of samples to ensure its robustness.

Table S1. neural network configurations with tanh and ReLU activations

dimensionality	Network architecture	Activation	Training epochs
2	2 – 100 – 100 – 1	Tanh	4000

2	2 – 100 – 100 – 1	ReLU	4000
2	2 – 30 – 40 – 30 – 40 – 1	Tanh	4000
2	2 – 40 – 30 – 20 – 30 – 1	ReLU	4000

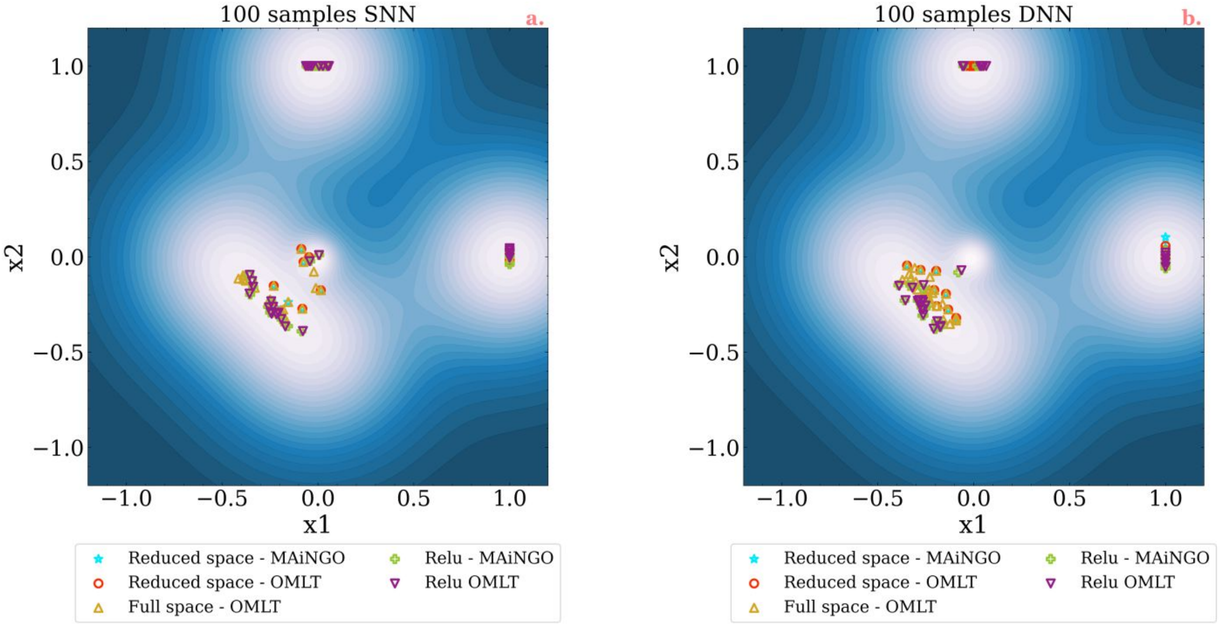

Figure S1. Visualizing the effect of sampling re-initialization on the variability of optimum solution for a) wider neural network vs b) deeper neural network with 100 samples

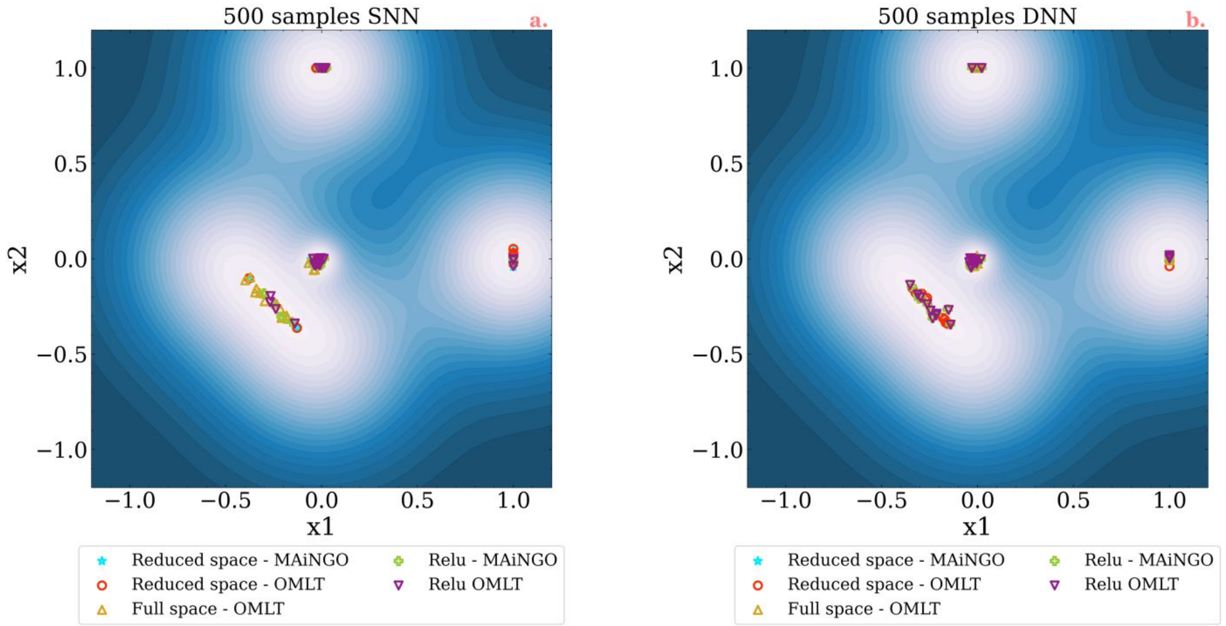

Figure S2. Visualizing the effect of sampling re-initialization on the variability of optimum solution for a) wider neural network vs b) deeper neural network with 500 samples

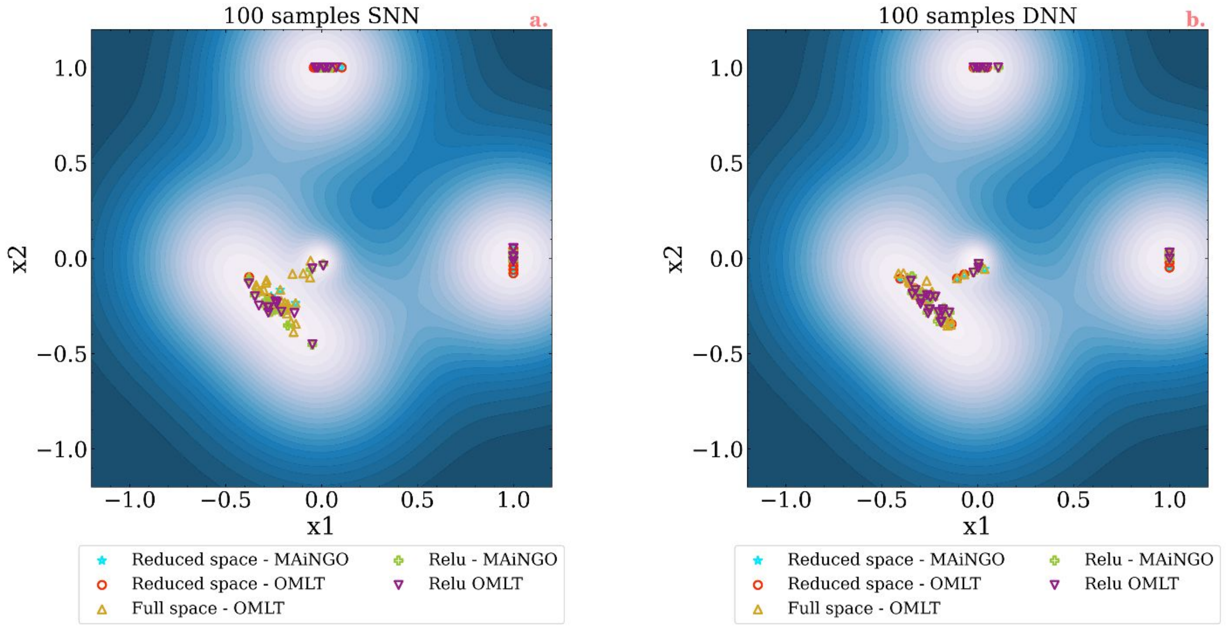

Figure S3. Visualizing the effect of sampling re-initialization on the variability of optimum solution for a) wider neural network vs b) deeper neural network with 100 samples and 7500 epochs

2. Effect of hybrid modeling on solution variability

The inclusion of hybrid modeling (HM) enhances the robustness of the model, resulting in lower variability in the optimized solution. However, it is important to recognize that the low-fidelity (LF) model can be nonlinear, as observed in this case, which complicates the MINLP formulation when using ReLU structures. This complexity necessitates the use of an MINLP solver, such as BARON (version 24.5.8) [1], to find the solution, thereby increasing the computational time required. A detailed analysis of the MINLP formulations, not included in the main text, is provided in Figure S5. In most repetitions, the MINLP solver could not converge within the specified time limit of 3,600 seconds for optimization. This time limit was set to allow for a fair comparison with the other methods used in the study. The relative error with true optimum and the time requirements are shown in Figure S5.

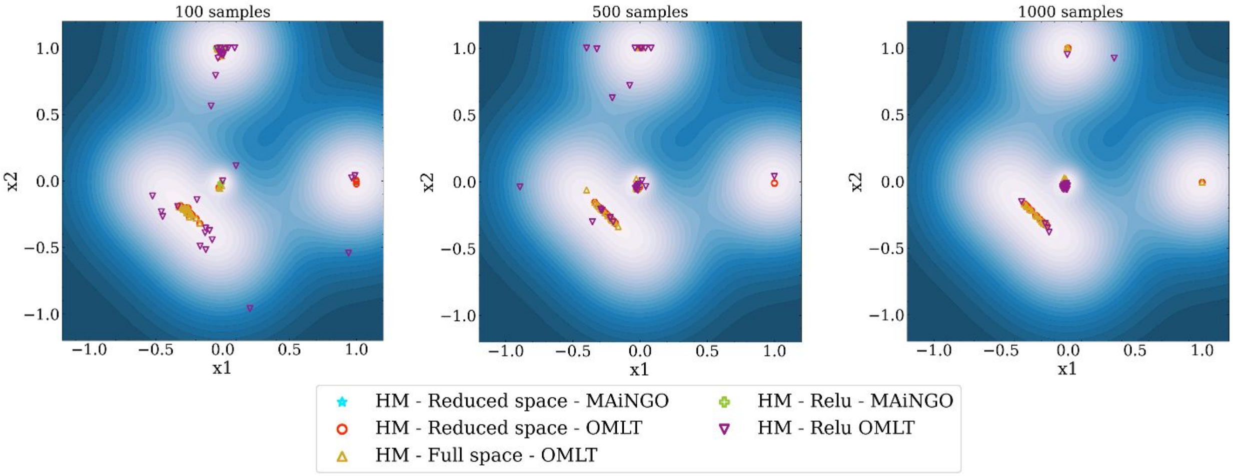

Figure S4. Visualizing the effect of sampling re-initialization on the variability of optimum solution for a-priori samples surrogate based optimization

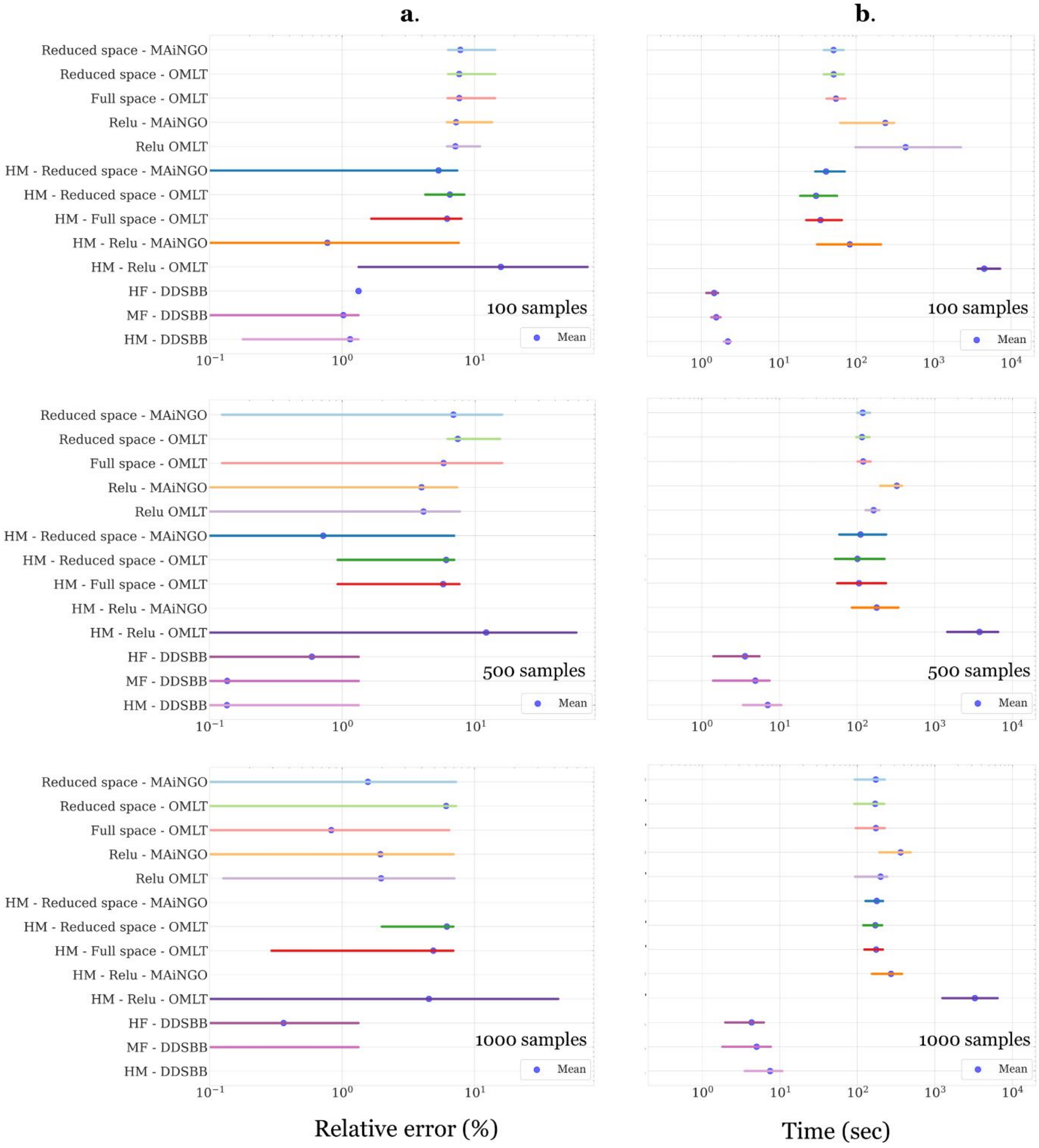

Figure S5. a) The relative error of the discovered solution with respect to the true optimum. b) The time requirements for modeling and optimization, for a-priori samples surrogate-based optimization and adaptive-sampling-based optimization with black-box and HM methods.

3. Tabulated information of results

To enhance the manuscript's comprehensiveness and numerical results, we provide tabulated information for the two mathematical case studies (Multigauss and Rastrigin function) to offer a more detailed comparison and facilitate easier reference for interested readers. Below in Tables S2, S3 and S4 we summarize the results of Multigauss function. In Tables S5, S6 and S7, we summarize the results for Rastrigin function case study.

Table S2. Maximum, minimum and average values of error and time for all the methods - 100 samples Multigauss case study

Tool	Maximum error (%)	Minimum error (%)	Average error (%)	Variance in error (%)	Maximum time (sec)	Minimum time (sec)	Average time (sec)
Reduced space - MAiNGO	14.329	6.304	7.801	2.689	68.611	38.233	51.031
Reduced space - OMLT	14.329	6.304	7.661	2.428	69.109	38.107	51.2
Full space - OMLT	14.329	6.251	7.656	3.186	72.48	41.236	54.633
Relu - MAiNGO	13.564	6.185	7.246	1.802	308.078	61.879	237.226
Relu OMLT	10.999	6.184	7.164	0.948	2251.911	98.046	434.283

HM - Reduced space - MAiNGO	7.402	0.046	5.349	8.737	71.182	29.516	40.812
HM - Reduced space - OMLT	8.375	4.238	6.506	0.461	56.531	18.925	30.32
HM - Full space - OMLT	7.949	1.656	6.2	1.159	65.265	22.603	34.551
HM - Relu - MAiNGO	7.595	0.002	0.773	4.947	208.731	31.202	82.546
HM - Relu - OMLT	71.466	1.329	15.793	345.759	7208.91	3698.574	4493.995
HF - DDSBB	1.329	1.329	1.329	0.0	1.65	1.157	1.469
MF - DDSBB	1.329	0.079	1.022	0.213	1.777	1.347	1.561
HM - DDSBB	1.329	0.178	1.148	0.12	2.418	1.952	2.209

Table S3. Maximum, minimum and average values of error and time for all the methods - 500 samples Multigauss case study

Tool	Maximum error (%)	Minimum error (%)	Average error (%)	Variance in error (%)	Maximum time (sec)	Minimum time (sec)	Average time (sec)
Reduced space - MAiNGO	15.926	0.124	6.845	11.028	147.148	100.056	118.078
Reduced space - OMLT	15.373	6.181	7.383	2.461	145.56	97.651	115.878
Full space - OMLT	15.931	0.124	5.767	17.322	149.875	101.206	119.763
Relu - MAiNGO	7.316	0.051	3.946	6.833	380.718	198.224	325.247
Relu OMLT	7.673	0.066	4.081	6.524	194.219	128.638	163.443
HM - Reduced space - MAiNGO	6.937	0.001	0.718	4.429	237.784	59.024	110.599

HM - Reduced space - OMLT	6.957	0.921	6.047	1.446	226.452	51.853	101.204
HM - Full space - OMLT	7.613	0.921	5.725	1.729	236.484	55.271	105.884
HM - Relu - MAiNGO	0.04	0.0	0.017	0.0	340.999	85.235	178.743
HM - Relu - OMLT	57.532	0.075	12.083	226.397	6580.458	1451.227	3770.603
HF - DDSBB	1.329	0.0	0.591	0.432	5.533	1.408	3.6
MF - DDSBB	1.329	0.0	0.136	0.164	7.488	1.391	4.898
HM - DDSBB	1.329	0.0	0.136	0.164	10.595	3.389	7.075

Table S4. Maximum, minimum and average values of error and time for all the methods - 1000 samples Multigauss case study

Tool	Maximum error (%)	Minimum error (%)	Average error (%)	Variance in error (%)	Maximum time (sec)	Minimum time (sec)	Average time (sec)
Reduced space - MAiNGO	7.242	0.003	1.563	7.721	225.741	91.851	172.299
Reduced space - OMLT	7.243	0.067	6.098	4.012	221.661	90.997	169.414
Full space - OMLT	6.424	0.003	0.827	2.881	226.107	94.531	173.27
Relu - MAiNGO	6.962	0.07	1.947	4.141	487.231	191.758	359.832
Relu OMLT	7.067	0.126	1.969	4.453	242.94	92.7	198.758
HM - Reduced space - MAiNGO	0.085	0.0	0.013	0.0	214.338	126.763	177.06
HM - Reduced space - OMLT	6.925	1.988	6.173	0.7	207.843	118.793	170.208

HM - Full space - OMLT	6.929	0.292	4.883	4.649	212.43	123.014	174.499
HM - Relu - MAiNGO	0.042	0.0	0.009	0.0	376.788	153.533	271.262
HM - Relu - OMLT	42.915	0.037	4.518	57.762	6438.029	1247.856	3285.066
HF - DDSBB	1.329	0.0	0.361	0.313	6.19	1.967	4.31
MF - DDSBB	1.329	0.0	0.091	0.113	7.637	1.805	4.996
HM - DDSBB	0.014	0.0	0.001	0.0	10.701	3.504	7.459

Table S5. Maximum, minimum and average values of error and time for all the methods – Rastrigin case study, dimensionality = 2

Tool	Maximum error (%)	Minimum error (%)	Average error (%)	Maximum time (sec)	Minimum time (sec)	Average time (sec)
Reduced space - MAiNGO	214.229	0.0	34.774	2272.329	2190.439	2237.915
Reduced space - OMLT	229.289	0.0	100.226	2272.672	2191.406	2239.014
Full space - OMLT	229.278	0.0	41.836	2277.578	2196.349	2243.987
Relu - MAiNGO	0.823	0.0	0.25	2491.847	2282.264	2390.888
Relu OMLT	1.279	0.013	0.57	2263.801	2190.826	2236.222
HF - DDSBB	29.272	0.0	2.686	2.438	0.078	0.669
MF - DDSBB	73.814	0.0	4.095	6.299	0.077	2.246
HM - DDSBB	8.313	0.0	0.277	8.326	0.511	4.927

**Table S6.** Maximum, minimum and average values of error and time for all the methods – Rastrigin case study, dimensionality = 5						
Tool	Maximum error (%)	Minimum error (%)	Average error (%)	Maximum time (sec)	Minimum time (sec)	Average time (sec)
Reduced space - MAiNGO	4.207	0.125	1.219	8930.407	8403.82	8573.57
Reduced space - OMLT	369.031	0.368	246.721	8346.039	8291.978	8317.924
Full space - OMLT	446.157	0.47	101.87	8348.955	8295.211	8321.185
Relu - MAiNGO	87.195	1.991	28.221	11206.227	10774.305	11028.619
Relu OMLT	86.61	2.018	29.181	10639.361	10547.832	10585.414
HF - DDSBB	58.477	0.0	5.13	23.331	1.358	7.786
MF - DDSBB	73.685	0.0	2.995	29.989	1.699	11.196
HM - DDSBB	0.0	0.0	0.0	46.079	9.277	36.571

**Table S7.** Maximum, minimum and average values of error and time for all the methods – Rastrigin case study, dimensionality = 10						
Tool	Maximum error (%)	Minimum error (%)	Average error (%)	Maximum time (sec)	Minimum time (sec)	Average time (sec)
Reduced space - MAiNGO	1045.191	229.289	614.917	16212.965	14778.655	15841.613
Reduced space - OMLT	984.035	169.555	643.72	15035.477	14523.022	14803.271
Full space - OMLT	971.7	322.113	675.256	15038.702	14530.31	14814.643
Relu - MAiNGO	1125.554	447.22	748.131	15274.554	14884.633	15092.912
Relu OMLT	1054.788	637.704	855.198	27886.397	14169.186	16404.629
HF - DDSBB	306.874	0.0	82.837	71.967	46.997	62.855
MF - DDSBB	299.83	0.0	141.128	56.124	8.722	32.868
HM - DDSBB	302.005	0.0	216.903	115.004	94.694	103.936

4. Impact of LF Model Linearity and NN Activation Functions on the Type of Optimization Problem

The MFSM optimization formulation incorporates the LF model equation as a constraint. Consequently, the nature of the constraint depends on whether a linear or non-linear LF model is employed. Specifically, utilizing a linear LF model results in a linear constraint; a non-linear LF model leads to a non-linear constraint and a ReLU NN formulation could lead to a MILP constraint. The NN responsible for correcting the LF model error within the MFSM framework can be constructed using different activation functions. In this study, we employ both Tanh and ReLU activation functions, which lead to nonlinear or mixed-integer linear constraints, respectively as shown in Table S8.

Table S8: Impact of LF Model Linearity and NN Activation Functions on the Type of Optimization Problem.

**LF Model**	**NN Activation Function**	**Resulting Optimization Problem**
Linear	Tanh	Non-Linear Problem (NLP)
Linear	ReLU	Mixed-Integer Problem (MIP)
Non-Linear	Tanh	Non-Linear Problem (NLP)
Non-Linear	ReLU	Mixed-Integer Non-Linear Problem (MINLP)

5. Comparison with other adaptive simulation-based optimization solvers

The three examples provide evidence that the integration of adaptive sampling methods with hybrid modeling can enhance the efficiency of optimization processes in simulation optimization. To underscore the importance of this approach, it is essential to conduct a comparative analysis with other widely used optimization solvers designed for simulation or black-box optimization tasks. Specifically, we will evaluate four solvers referenced in [2]: Bayesian Optimizer, DIRECT Algorithm, Lipschitz Optimizer, and Particle Swarm Optimizer. Notably, the Particle Swarm Optimizer is a population-based solver, whereas the remaining solvers employ sequential-model based strategies and adaptively explore the search space for optimum. Building on our previous analysis, we evaluate the time requirements and the impact of variations in sampling design on the optimal solution by repeating the optimization of the two dimensional case study - *multigauss*, thirty times. For consistency, we use the default parameters for each solver and set the maximum number of samples at 500. This approach allows us to gather comprehensive data on the performance and efficiency of the optimization processes under standardized conditions.

Figure S6 illustrates the results on solution variability with sampling re-initialization, indicating minimal variation in the optimum solutions across all methods. This consistency suggests that the solvers are effective in locating solutions. To delve deeper, Table S9 presents the relative error and time requirements for all methods across thirty repetitions. Although the relative errors are low, the time requirements plot reveals significant differences among the solvers. Specifically, the Lipschitz optimizer requires the most time, followed by the Bayesian optimizer, whereas the DIRECT algorithm and Particle Swarm Optimizer require the least time. However, a detailed analysis of the sampling requirements reveals a different

perspective. Despite a limit of 500 high-fidelity (HF) samples, the collection of data on the best solutions occurred before the sampling limits were reached.

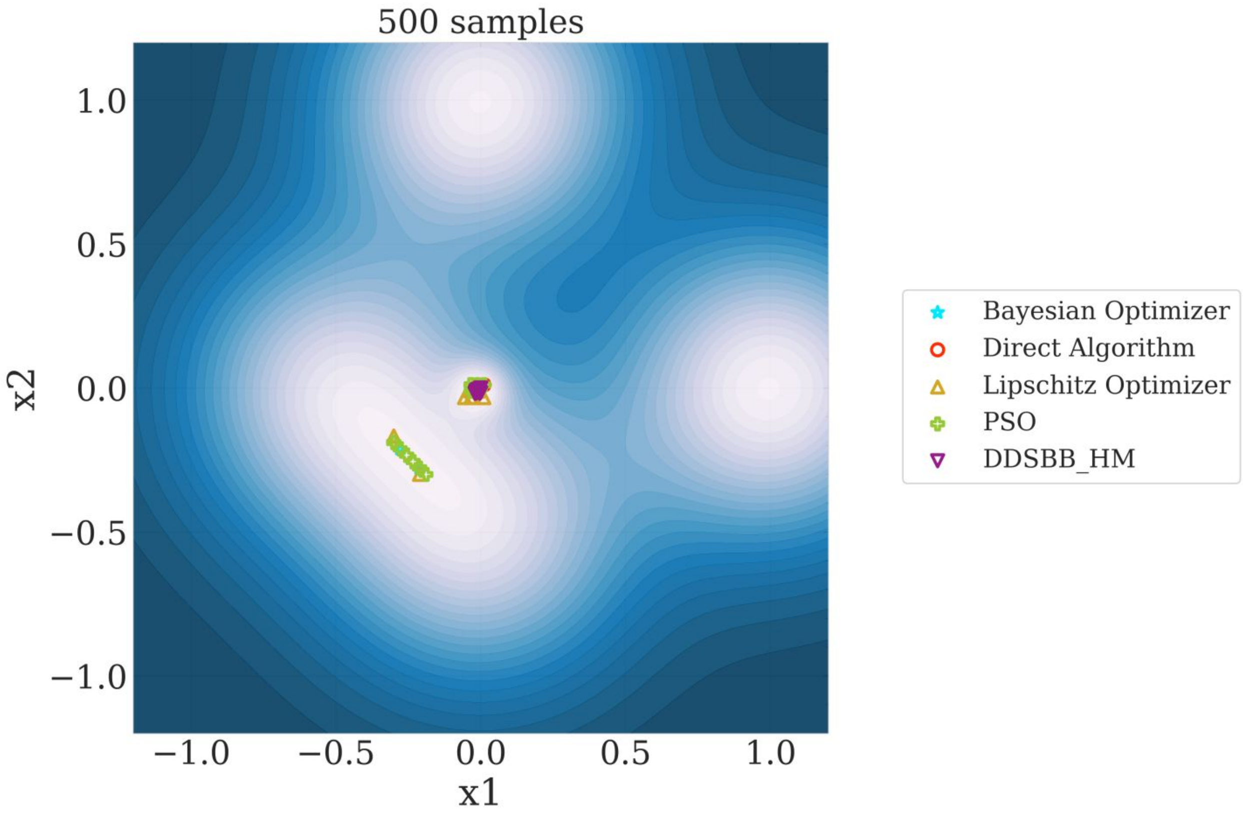

Figure S6. Visualizing the effect of sampling re-initialization on the variability of optimum solution for sampling based methods

Table S9. Maximum, minimum and average values of error and time for all the solvers

Tool	Maximum Error (%)	Minimum Error (%)	Average Error (%)	Maximum Time (sec)	Minimum Time (sec)	Average Time (sec)	Average Samples
Bayesian Optimizer	6.189	0.041	5.369	90.145	65.664	76.028	491
Direct Algorithm	4.478	4.478	4.478	0.085	0.049	0.058	500

Lipschitz Optimizer	6.339	0.041	1.179	290.656	276.159	280.082	205
PSO	6.193	0.041	3.264	0.047	0.034	0.039	381
DDSBB_HM	1.329	0.001	0.233	8.102	1.967	4.934	232

Figure S7 illustrates the relationship between sampling requirements and the evolution of the optimum solution as a function of the number of samples. For practical applications, the ideal solver would be one that minimizes the use of HF samples and time while delivering consistent solutions. When considering all these factors, hybrid modeling integrated DDSBB has the optimal performance across these criteria. This underscores the advantage of integrating hybrid modeling with the adaptive sampling solvers.

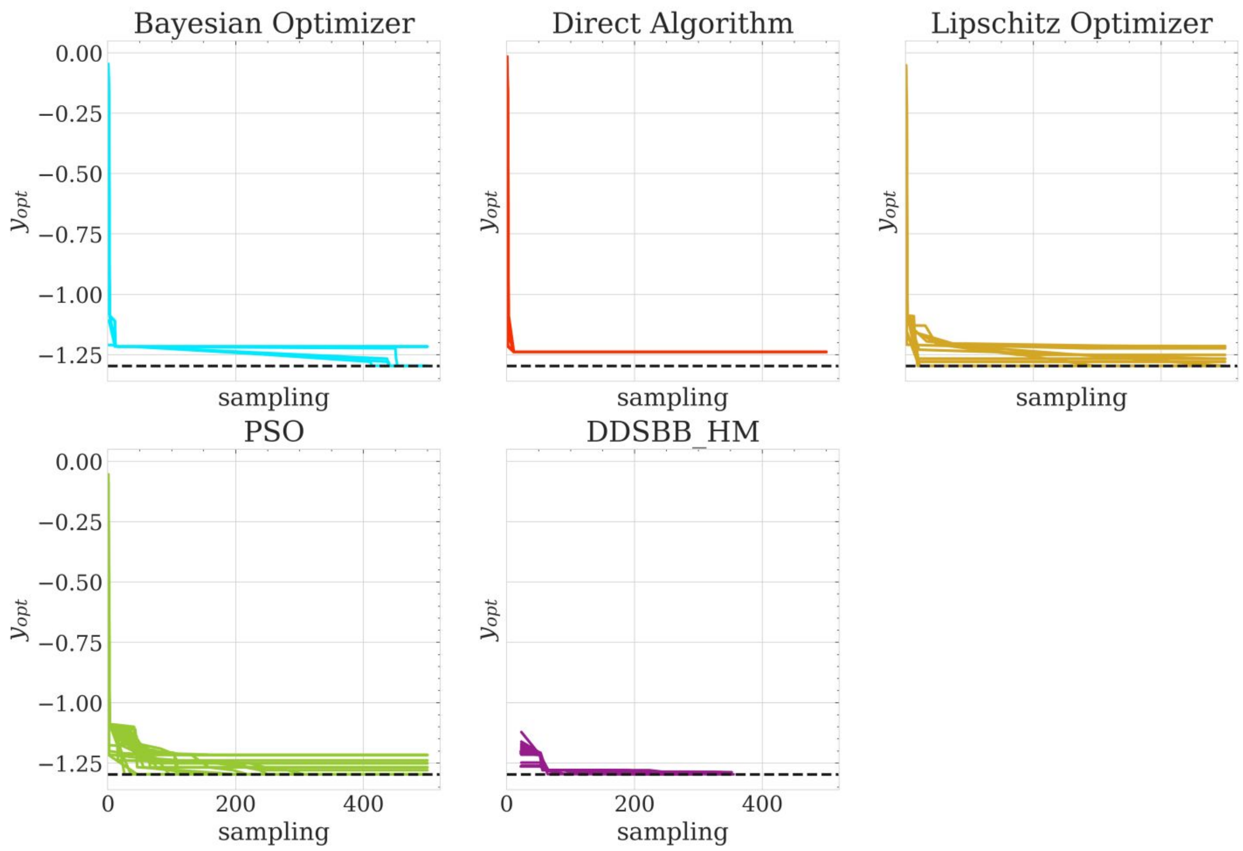

Figure S7. Evolution of optimum solution found with sample number, shown in dotted black line is the true optimum

6. TVSA optimization formulation

The TVSA was modeled by Min et al [3] with partial differential-algebraic system of equations. The TVSA model used in the optimization is the Min et al's model with modified geometry.

Simulation inputs and outputs:

$$[q_{ads}, q_{des}, TEU, dP] = TVSA(T, RH, u_{steam}, q_{ini}, t_{ads}, t_{des}) \quad \text{Eq (S1)}$$

Main equations in TVSA:

Mass Balance:

$$\varepsilon_m\left(\frac{\partial C_i}{\partial t} + \frac{\partial(v_g C_i)}{\partial z}\right) = \varepsilon_m D_g \frac{\partial^2 C_i}{\partial z^2} - S_i \quad \text{Eq (S2)}$$

$$S_{CO_2} = (1 - \varepsilon_m)\rho_c \frac{\partial q_{CO_2}}{\partial t} \quad \text{Eq (S3)}$$

$$S_{N_2} = 0 \quad \text{Eq (S4)}$$

Energy Balance:

$$\rho_g C_{p,g}\left(\frac{\partial T_g}{\partial t} + \frac{\partial(v_g T_g)}{\partial z}\right) = \lambda_g \frac{\partial^2 T_g}{\partial z^2} - AQ_{conv} \quad \text{Eq (S5)}$$

$$\rho_c C_{p,c} \frac{\partial T_c}{\partial t} = \lambda_c \frac{\partial^2 T_c}{\partial z^2} + AQ_{conv} + Q_{gen,CO_2} + Q_{gen,H_2O} + AH_{vap} \quad \text{Eq (S6)}$$

$$Q_{conv} = h(T_g - T_c) \quad \text{Eq (S7)}$$

$$Q_{gen,CO_2} = \Delta H_{CO_2} \rho_c \frac{\partial q_{CO_2}}{\partial t} \quad \text{Eq (S8)}$$

Momentum Balance:

$$u_g = -\frac{dP}{dz}\frac{1}{\mu_g}\left[\frac{r_o^2 - 3r_{fs}^2}{8} + \frac{r_{fs}^4}{2\left(r_{fs}^2 - r_o^2\right)} ln\left(\frac{r_{fs}}{r_o}\right)\right] \quad \text{Eq (S9)}$$

The Technoeconomic Analysis (TEA) Model uses the simulation results of TVSA model to calculate the cost (OCNC) as the objective function of this optimization. The TEA model used was from Holmes et al [4].

TEA:

$[OCNC_i, NC_i] = TEA(q_{ads,i}, q_{des,i}, t_{ads,i}, t_{des,i}, TEU_i, dP_i)$ for cycle i

$OCNC$: Operational cost per net CO_2 captured [$/net-$CO_2$ captured]

NC: Net CO_2 Captured

q_{ads}: Average CO_2 loading on the fiber at the end of adsorption step [mol-CO_2/kg-fiber]

q_{des}: Average CO_2 loading on the fiber at the end of desorption step [mol-CO_2/kg-fiber]

t_{ads}: Adsorption duration [sec]

t_{des}: Desorption duration [sec]

TEU: Thermal energy used [J/mol-CO_2 captured]

dP: Pressure drop of column [Pa]

Optimization problem of TVSA:

$$\min_{A_i,D_i} OCNC_{avg} \quad \text{Eq (S10)}$$

$$s.t.\begin{cases} [q_{ads,i},q_{des,i},TEU_i,dP_i] = TVSA(T_i,RH_i,u_{steam,\,i},t_{ads,i} = A_i,t_{des,i} = D_i) \\ [OCNC_i,NC_i] = TEA(q_{ads,i},q_{des,i},u_{steam,\,i},t_{ads,i} = A_i,t_{des,i} = D_i,TEU_i,dP_i) \\ q_{ini,i} = q_{end,i-1} \\ q_{ini,0} = q_0 \\ 2000 \leq A_i \leq 8000\ sec \\ 1500 \leq D_i \leq 3000\ sec \\ OCNC_{avg} = \frac{\sum_i NC_i \times OCNC_i}{\sum_i NC_i} \end{cases} \quad \text{Eq (S11)}$$

For the current manuscript, $i\ = \{1,2,3\}$

7. Comparing surrogate options for Low-fidelity models

To represent the LF simulation, we selected an SVR model due to its lower complexity and adequate accuracy. Our analysis demonstrated that the SVR model effectively captures the LF simulation, achieving R^2 scores exceeding 0.92 for the training set and 0.83 for the testing set. A rigorous analysis should include evaluating additional surrogate models to identify the optimal representation of the LF simulation, which involves hyperparameter tuning. We did not do this because the LF model does not need to be perfectly accurate by definition. To address errors arising from the surrogate LF model, we employed a NN to correct

these errors using the MFSM approach. At this stage, hyperparameter tuning was conducted to determine the best architecture and corresponding hyperparameters for the NN. While a more complex surrogate model might better fit the LF data, it would necessitate extensive hyperparameter tuning. Additionally, since the LF model equation serves as a constraint in the optimization formulations within the MFSM, a more complex model would result in a significantly larger nonlinear formulation. Therefore, it is essential to balance the size and complexity of the LF model and the error-correcting NN.

We present the performance of different surrogate models using the same LF simulation data for both training and testing sets. We utilized scikit-learn (sklearn) for building the surrogate models and tuning their parameters. A dataset comprising 5,000 LF data points was divided into 3,000 training points and 2,000 testing points. We evaluated SVR, Gaussian Process Regression (GPR), and NN models as candidates for the surrogate model. Figure S8 illustrates the performance of these surrogate models on both the training and testing sets.

For the SVR model, we tuned the regularization parameter C, gamma, and epsilon. For the GPR model, we tuned the kernel constant and length scale. For the NN model, we varied the number of nodes from 5 to 50, the number of layers from 1 to 2, and tested both tanh and ReLU activation functions. Among the three models considered, the SVR model exhibited the smallest difference in performance between the training and testing sets, indicating better generalization.

A similar analysis was performed for the extractive distillation case study. This case study consisted of 5 inputs and one output and treated as black-box simulation. In this case too, the simulation is continuous with respect to input and output space but is infeasible at some points due to infeasiblites on purity requirements. For this case, the SVR and NN models had a similar accuracy although the SVR model had

fewer parameters. Hence we chose SVR as the surrogate option for the LF model. The train and test accuracies are shown in Figure S9.

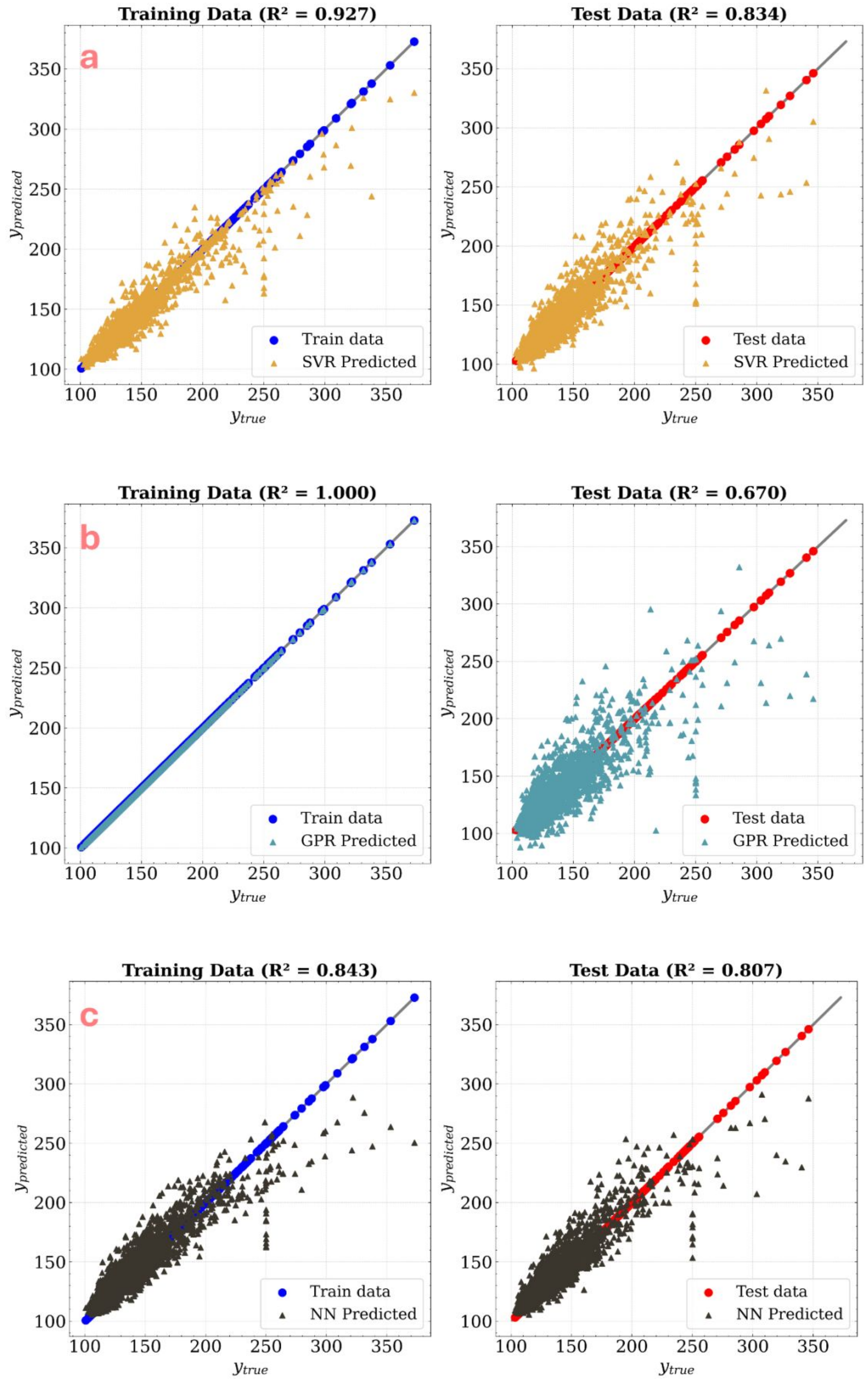

Figure S8: Performance of a) SVR, b) GPR and c) NN surrogate models used to fit the LF TVSA simulation on the train and test data sets.

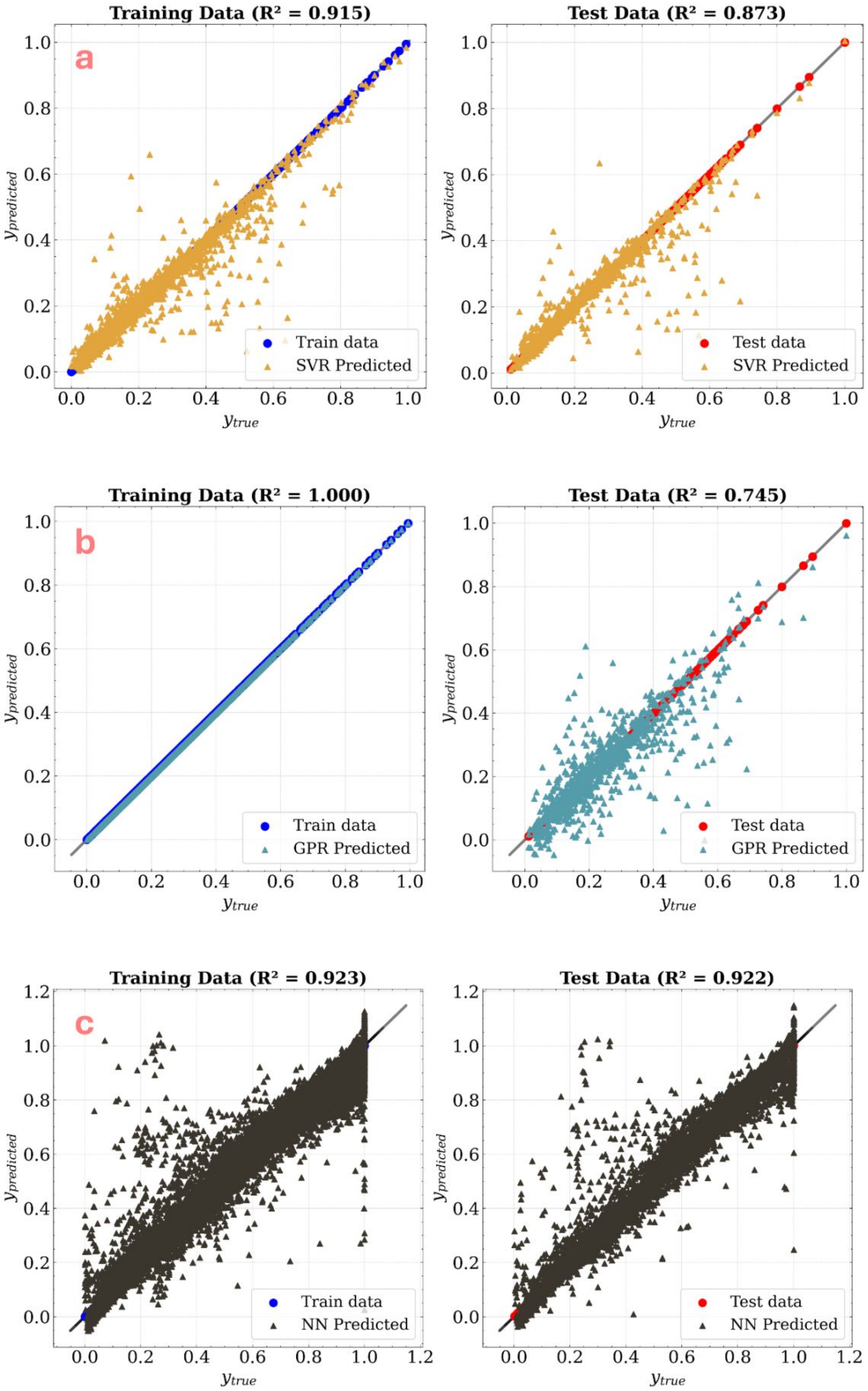

Figure S9: Performance of a) SVR, b) GPR and c) NN surrogate models used to fit the extractive distillation HF simulation on the train and test data sets.

While Polynomial Regression can be very effective for capturing specific types of relationships and should be used when appropriate, in this paper we limit our comparisons to nonparametric ML models, which utilize kernel/activation functions to implicitly handle high-dimensional mappings due to their flexibility.

8. Non-linearity and performance LF and NN models in MFSMs

Identifying the mismatches between the MFSM fit and the true function profile provides insight into model performance. Second, comparing the derivative profiles of the MFSM and the true function offers additional validation.

To test this, we selected the Multigauss case study from the manuscript and conducted experiments by fitting the LF model and the subsequent MFSM using 500 samples, repeating the process five times with re-initializations. The fits obtained for one of these cases are presented in Figure S10. Specifically, we show results for:

- NN: direct black-box fit of the HF data using a Neural Network (NN),
- HM with LF-SVR: MFSM or Hybrid Model (HM), which is a composite of a LF surrogate using Support Vector Regression (LF-SVR) and a NN correction model. For this case, we show both the profiles of the LF model and the overall HM model.
- HM with LF-MG: MFSM or Hybrid Model (HM), which is a composite of a LF Multigauss (LF-MG) function (described in the manuscript) and a NN correction model. For this case, we show both the profiles of the LF model and the overall HM model.

It is important to note that all of the structures contain the same number of overall fitted parameters and were trained with the same HF data. Specifically, we employed the same NN architecture identified as optimal in the manuscript (2-100-100-1) configuration.

The results show that the SVR-based LF model captures the general non-linear behavior of the true function, although its accuracy is limited when fewer than 75 support vectors are used. The LF-MG model also captures some valleys and peaks of the true function but is not as accurate as HF data. In both cases, the overall HM structure is able to provide an accurate and smooth response.

When a NN is trained directly on the data, its profile is noticeably less smooth compared to the true function. Incorporating the SVR output into the NN through the MFSM/HM formulation leads to an improved fit and a smoother function. This improvement can be attributed to the SVR capturing partially the system's general nonlinear form, allowing NN to focus on modeling any remaining non-linear components.

Additionally, we examined a case where the LF model uses the LF-Multigauss function instead of SVR. The LF-Multigauss function better captures the true function profile, particularly around local solutions. In this case, passing the LF output to the same NN architecture also results in a higher-quality fit. The smoother and more accurate profiles are consistent for the LF-Multigauss function, providing a superior initial approximation, enabling the NN to focus on refining residual non-linearities.

This further strengths the hypothesis that even with varying accuracy of LF models, the HM structures can better learn the overall response.

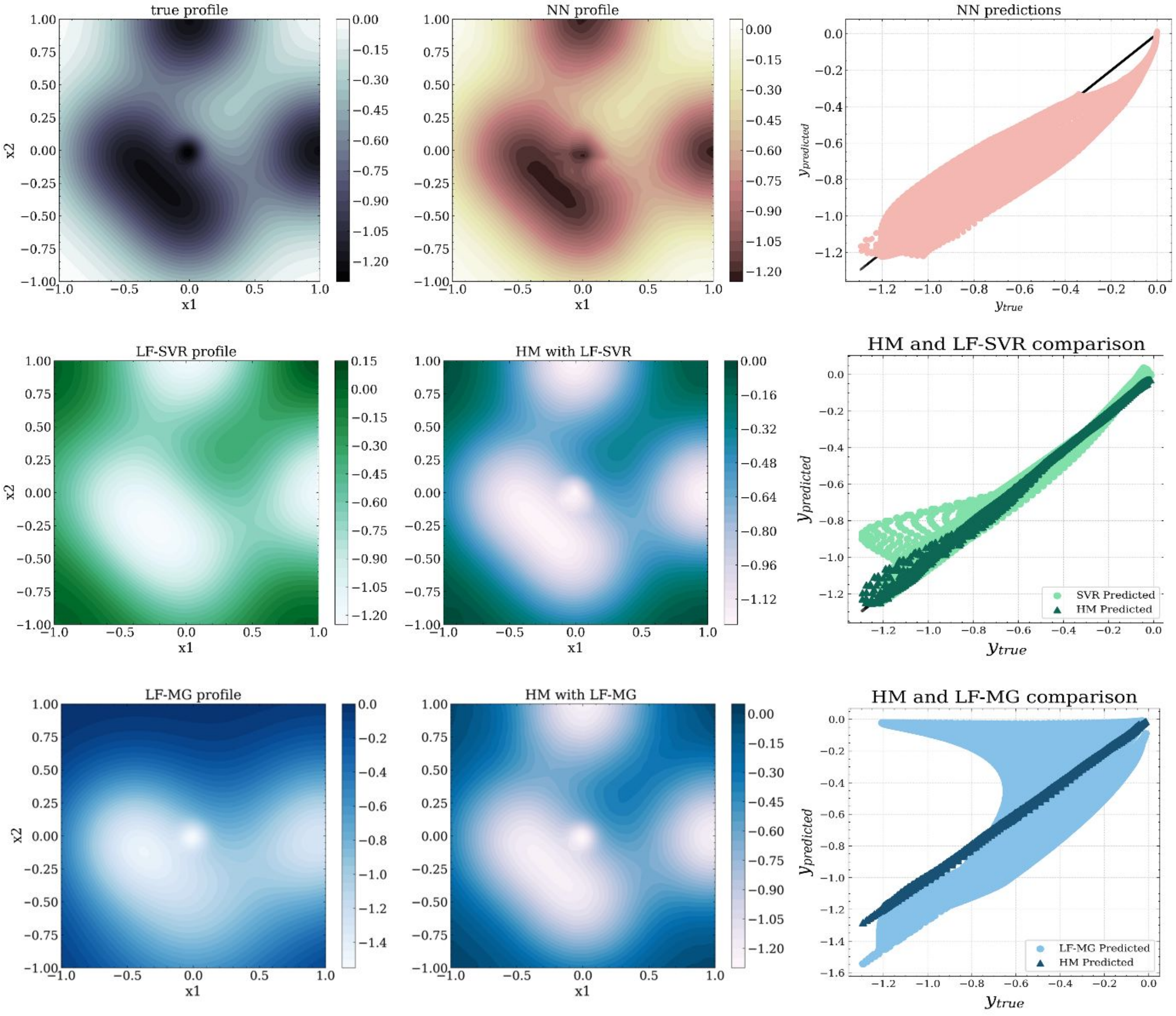

Figure S10: LF, BB- NN and HM fit comparison with true function profile. The top panel corresponds to black-box NN fit shown in yellow contour. True function profile shown in black and white contour. In the second pane, LF -SVR model profile shown in green contour. MFSM profile with LF-SVR blue-green contour. LF-Multigauss (MG) profile and the corresponding MFSM with LF-Multigauss function is shown in blue contours. Also shown in the last figure of each panel, is the parity plot of these model predictions.

To further understand and visualize the MFSM fit, we compared it against the true function profile and analyzed the error (mismatch) as a function of the variables x_1 and x_2. Ideally, this difference should be

zero for a perfect fit. We also examined the derivatives mismatch profiles for both the MFSMs and the black-box NN model to evaluate the smoothness of the resulting functions.

This analysis revealed two key insights. First, as observed from the error profiles (with attention to the scales), the MFSM utilizing the LF-Multigauss (LF-MG) function exhibited the smallest variation (smoother without any noisy errors and derivative errors), followed by the MFSM with the LF-SVR model, and lastly, the black-box NN model, which showed the highest variation. A similar trend was noted in the derivatives mismatch profiles, where the black-box NN model displayed larger deviations. This indicates that models with less noisy derivatives, such as those using the LF-MG function, are less prone to local minima.

Second, this implies that when these models are optimized using deterministic solvers, the presence of noisy derivatives in the black-box NN model increases the likelihood of encountering local optima. This can result in longer optimization times and potentially sub-optimal solutions. These findings reinforce the conclusions presented in the manuscript, further supporting the robustness of our approach. Hence it can be said that MFSM structures help in improving the surrogate fit, smoothness and robustness, and potentially help in improving the convergence to global solutions.

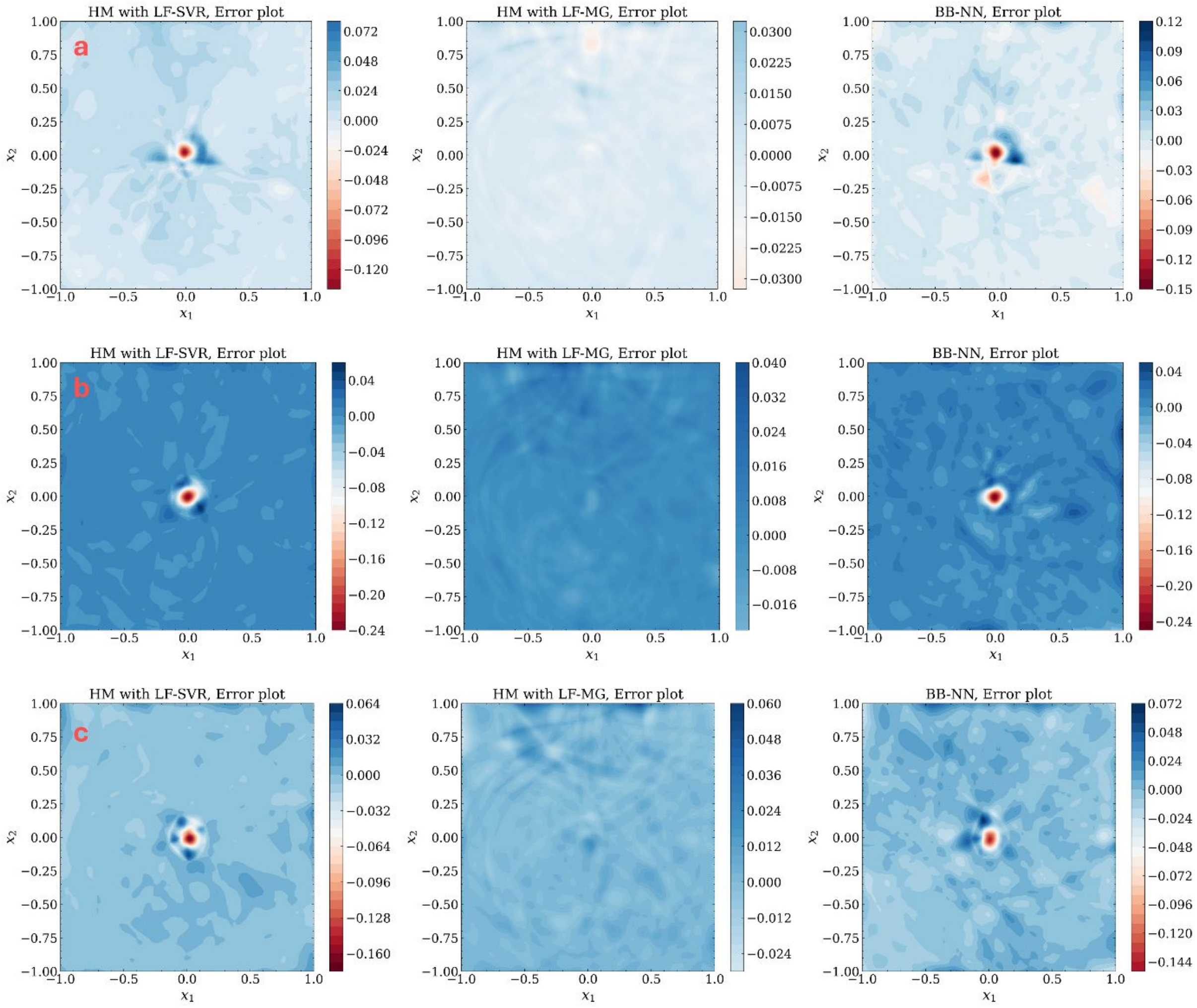

Figure S11: BB- NN and MFSMs mismatch comparison with true function profile. The three panels a, b, and c correspond to three re-initialization experiments. (please check the scales)

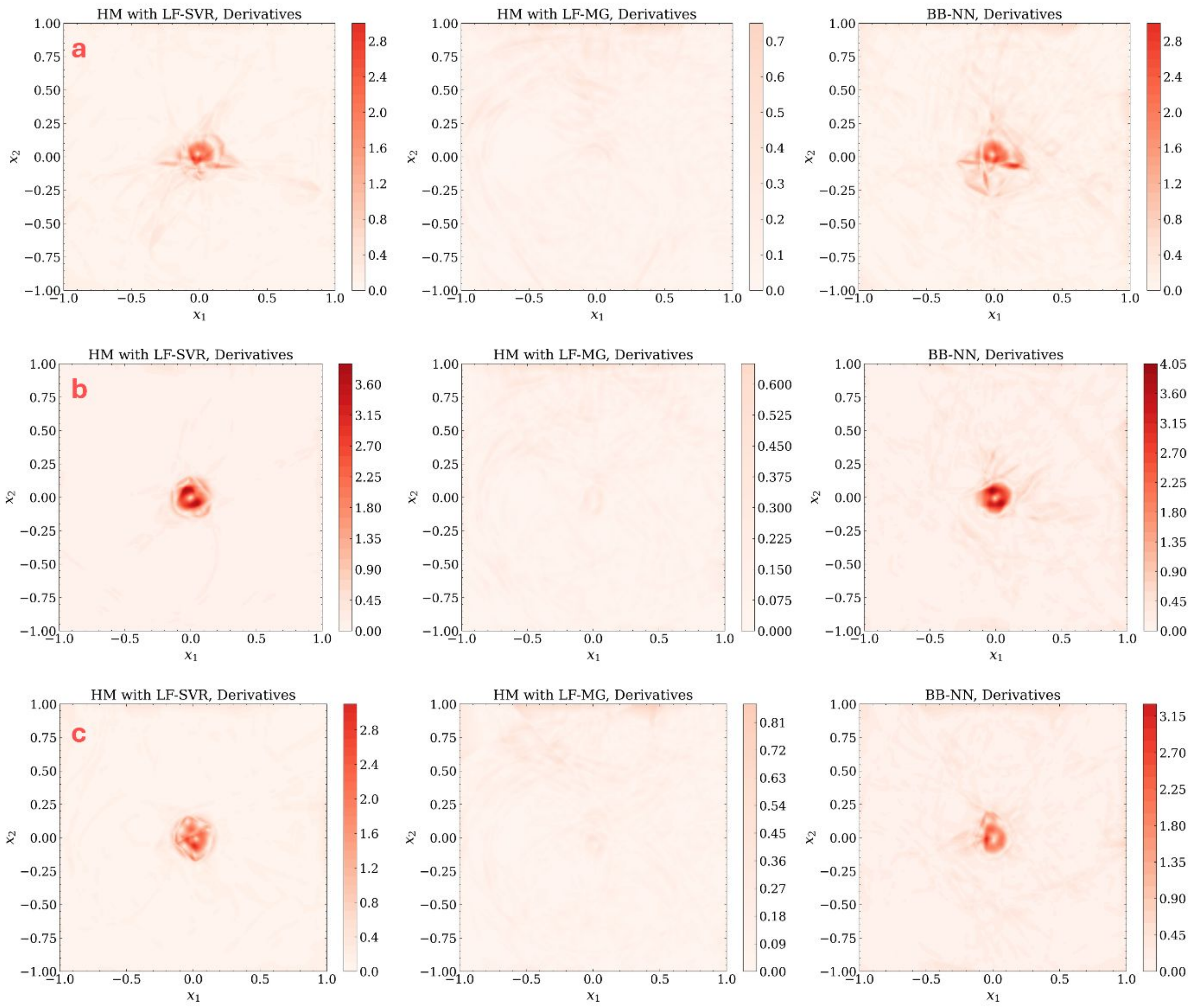

Figure S12: BB- NN and MFSMs derivative mismatch comparison with true function profile. The three panels a, b, and c correspond to three re-initialization experiments. (please check the scales)

Relevant Abbreviations:

HF – High-fidelity

LF – Low-fidelity

MF – Multi-fidelity

ML – Machine learning

HM – Hybrid modeling

MFSM – Multi-fidelity surrogate model

OMLT - Optimization and Machine Learning Toolkit

MAiNGO - McCormick-based Algorithm for mixed-integer Nonlinear Global Optimization

DDSBB – Data-driven spatial branch-and-bound

NN – Neural network

RS – Reduced space

FS – Full space

ReLU - Rectified Linear Unit

SVR – Support vector regression

GPR – Gaussian process regression

UB – Upper bound

LB – Lower bound

MG – Multi-gauss

LHS - Latin hypercube sampling

MSE - Mean squared error

MILP – Mixed integer linear program

NLP – Non-linear program

MINLP - Mixed integer non-linear program

MIP - Mixed-integer programming

IPOPT - Interior Point Optimizer

TVSA - Temperature vacuum swing adsorption

OCNC - Operational cost per net CO_2 captured

DAC – Direct air capture

REFERENCES

(1) Ryoo, H. S.; Sahinidis, N. V. A branch-and-reduce approach to global optimization. *Journal of global optimization* **1996**, *8*, 107-138.

(2) Blanke, S. *Gradient-Free-Optimizers: Simple and reliable optimization with local, global, population-based and sequential techniques in numerical search spaces.* 2020. https://github.com/SimonBlanke/Gradient-Free-Optimizers (accessed.

(3) Min, Y. J.; Kim, J.; Jones, C. W.; Realff, M. J. Model-Based Energy and Cost Analysis of Direct Air Capture Using ePTFE-Based Laminate-Structured Gas–Solid Contactors. *ACS Sustainable Chemistry & Engineering* **2024**.

(4) Holmes, H. E.; Lively, R. P.; Realff, M. J. Defining targets for adsorbent material performance to enable viable BECCS processes. *JACS Au* **2021**, *1* (6), 795-806.
